# Design Principles for Surface-Passivating Ligands of Cesium Lead Halide Perovskite Nanocrystals in the Strongly Quantum-Confined Regime

**DOI:** 10.1021/acs.chemmater.5c03187

**Published:** 2026-03-19

**Authors:** Seungjun Cha, Courtney Brea, Aaron Malinoski, Chen Wang, Guoxiang Hu

**Affiliations:** † School of Materials Science and Engineering, 1372 Georgia Institute of Technology, Atlanta, Georgia 30332, United States; ‡ Department of Chemistry and Biochemistry, Queens College, 14772 City University of New York, New York, New York 11367, United States; § The Graduate Center, City University of New York, New York, New York 10016, United States; ∥ School of Chemistry and Biochemistry, Georgia Institute of Technology, Atlanta, Georgia 30332, United States

## Abstract

Passivation of surface defects of cesium lead halide (CsPbX_3_, X = Cl, Br, I) nanocrystals is crucial to improving the stability and photoluminescence of these materials for further optoelectronic applications. Many ligands have been examined for surface passivation; however, a ligand design principle for improved photoluminescence quantum yield (PLQY) is still not available. Here, we report a combined computational and experimental study to systematically investigate 27 commercially available ligands and develop foundational guidelines. Using first-principles density functional theory, we calculated the binding energy of the ligands on the CsPbBr_3_ nanocrystal. We find a volcano relationship between ligand binding energy and the experimental PLQY, which reveals the negative impact of overly strong binding energy. We further perform electronic structure analysis and time-resolved optical spectroscopy to reveal that these strong-binding ligands can withdraw more electrons from the surface and induce trap states within the bandgap. With this, we develop a design principle for the PLQY of CsPbBr_3_ nanocrystals, highlighting the importance of the ligand binding energy comparable to that of the native halide species. We further applied this design principle to quantum-confined CsPbCl_3_ and CsPbI_3_ nanocrystals, and our computational predictions have been successfully validated by experiments.

## Graphical abstract

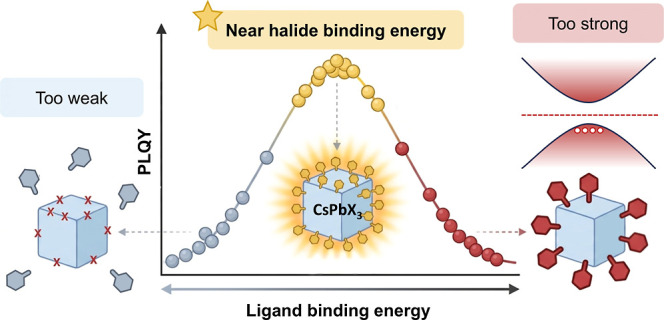

## 1 Introduction

Halide perovskite nanocrystals have gained significant attention as next-generation optoelectronic materials due to their excellent optical properties, including narrow emission spectra and easily tunable emission wavelengths.^1^ Especially, the all-inorganic cesium lead halide perovskite (CsPbX_3_, X = Cl, Br, and I) nanocrystals have emerged rapidly in the research community, following the first report of their colloidal synthesis via the hot injection method.^2^ The facile and low-cost nature of solution-based production has paved the way for a range of optoelectronic applications such as solar cells,^3^ light-emitting diodes,^4^ lasers,^5^ photodetectors,^6^ and single-photon sources.^7^ Their superior optical properties and intriguing surface chemistry also make them promising candidates for developing novel photocatalysts.^8^

Despite these promises, recent computational and experimental studies continue to reveal that surface defects of CsPbX_3_ nanocrystals pose a significant challenge for further practical uses. While bulk CsPbX_3_ (X = Br and I) perovskites are generally accepted as defect-tolerant, with the defect-induced trap states forming within or near the band edges (i.e., shallow traps) and remaining benign to optoelectronic properties,^9^ this tolerance diminishes in CsPbCl_3_ and perovskite nanocrystals in the strongly quantum-confined regime, which shifts the band edges. Energy levels of the localized trap states are relatively insensitive to the change of the halide component and the particle size, thus making them now reside deeper in the bandgap^10–12^ to facilitate nonradiative recombination, which prevents the near-unity photoluminescence quantum yield (PLQY). Due to the high surface-to-volume ratio of nanocrystals^13^ and the highly dynamic ligand shell,^14^ these deep traps are predominantly induced by surface defects, particularly halide vacancies (i.e., uncoordinated lead), which are favored by low formation energy compared to other types of defects in CsPbX_3_.^15,16^ Surface vacancies are also undesirable from the stability perspective as they essentially act as reactive sites for atmospheric oxygen and moisture, leading to degradation into nonperovskite phases.^16,17^ Therefore, passivating surface vacancies is an imperative task to simultaneously improve the PLQY and stability for practical applications.

Surface vacancies are readily produced from the highly dynamic equilibrium between bound and free states of the native oleate (OA) and oleylammonium (OlAm) capping ligands introduced during typical colloidal synthesis of CsPbX_3_ nanocrystals.^14^ The labile nature of these ligands is regarded as the leading cause of drops in PLQY after repeated cycles of purifications, during which the ligands are irreversibly lost and vacancies are left behind.^18,19^ Therefore, many experimental efforts have focused on ligand engineering strategies to employ ligands with strong binding for enhanced surface passivation.^13^ These include the usage of: (1) soft base ligand^15,20^ to improve binding overlap, considering Pb^2+^ is a soft acid according to the Hard and Soft Acids and Bases theory, (2) multidentate ligands, as they allow more stable coordination of lead due to the chelate effect,^21–23^ and (3) inorganic metal halides such as ZnBr_2_ ^24^ and AgBr,^25^ which directly fill the halide vacancies. These ligands can be either incorporated by direct synthesis (in situ) or by postsynthesis treatment via simple addition or ligand exchange procedures. While these ligands have been clearly demonstrated to improve PLQY and the surface stability, there have been no systematic comparisons of the ligands along with the design principle. In other words, it is not known whether certain ligands would provide better passivation than others, and if it is the case, what the underlying reasons are. The currently known guiding principle primarily emphasizes strengthening the binding affinity of the ligand; however, recent studies suggest that strong binding does not necessarily lead to improved PLQY. For example, strong ligand–core interactions can be accompanied by enhanced electronic coupling,^26,27^ which may introduce additional nonradiative recombination pathways. Under certain conditions, overly strong or highly reactive ligands can also promote undesirable phase transformations into nonluminescent phases by facilitating side reactions.^28,29^

Here, we combine first-principles density functional theory (DFT) calculations with experimental measurements to provide insights into design principles of surface-passivating ligands for improved PLQY of cesium lead halide perovskite nanocrystals. Through investigations of 27 different ligands on the CsPbBr_3_ nanocrystal in the strongly quantum-confined regime, we reveal a nonmonotonic relationship between the ligand binding energy and PLQY. Based on this, we propose a design principle for improving the PLQY of CsPbBr_3_ nanocrystals, emphasizing the important role of ligand binding energy relative to that of the native halide species. We show that the proposed principle can be further extended to the search for ligands that can passivate CsPbCl_3_ and CsPbI_3_ nanocrystals, as validated by experiments. Our work provides an effective guideline for screening a larger subspace of surface ligands for all halide compositions and sheds light on the role of charge distributions at the ligand–perovskite interface in PLQY improvement for practical optoelectronic applications.

## 2 Results and Discussion

### 2.1 Volcano Relationship between Ligand Binding Energy and PLQY

We prepared CsPbBr_3_ nanocrystals passivated by 27 commercially available ligands by colloidal synthesis and postsynthetic ligand exchange treatment. Across all samples, cubic morphology with an average diameter of 4 nm was observed, which is smaller than the reported Bohr-exciton diameter of CsPbBr_3_.^2^ Similarly, the first excitonic band at 458 nm (2.7 eV) was higher than that of the bulk, to corroborate that as-synthesized nanocrystals were in the quantum-confined regime. The PLQY of the as-synthesized perovskite nanocrystal strongly depends on the polarities of the solvents, as we reported earlier.^12^ In nonpolar solvents, such as cyclohexane, the intrinsic PLQY can reach 0.60 ± 0.05, similar to the reported values,^30^ but it decreases to 0.23 ± 0.06 when dissolved in toluene.^12^ We chose toluene as the solvent for collecting experimental data to better demonstrate the ligand effects. All passivated samples had subunity PLQY that varied significantly from 0.23 to 0.85, indicating the strong influence of ligands on the degree of deep trap passivation. Slight spectral shifts (2–5 nm) were observed after the passivation treatment for a few ligands, in particular, for zwitterion ligands, such as 4-ABS, while the bandgap transition remains a narrow energy distribution. We do not expect the spectral shifts to be due to PNC damage nor should they significantly affect the passivation. We speculate that the spectral shift can be attributed to two possible reasons: (1) introducing ligands slightly disturbs the metastable colloidal phase and causes a slight size change of the PNCs; (2) zwitterion ligands may generate an interfacial electric field that induces a Stark effect on the band gap transition.

Using first-principles DFT, we calculated the binding energies of the 27 ligands on the CsPbBr_3_ nanocrystal and correlated these energies to the experimentally measured PLQY (Table S1 provides a complete list of the binding energy and PLQY for the ligands). The calculations employed a slab model containing a methylammonium substituting a nearby Cs^+^ site, representing an ammonium-based cation commonly found on perovskite surface,^18,31^ and a single halide vacancy, a predominant defect type responsible for midgap states and exciton trapping.^11,15^ The passivating ligand was positioned at this vacancy site, and the binding energy was determined from the total energy difference between the ligand-bound and unbound configurations. For zwitterionic ligands, an additional Cs^+^ cation was removed for charge neutrality and enabled direct interaction between the ligand’s ammonium group and the surface, providing additional stabilization through hydrogen bonding. This model configuration and defect type are consistent with previous computational and experimental studies;^12,18,31^ further details are provided in the Supporting Information. As shown in Figure 1, a volcano relationship, instead of a monotonic relationship as thought previously, was observed between the ligand binding energy and PLQY. We found that ligands whose binding energies are closer to that of bromide (ΔE_Br_ = −2.01 eV, as indicated by the green dotted line), generally have higher PLQY. Ligands with larger or smaller binding energies have lower PLQY. The positive correlation between binding energy and PLQY on the left-hand side of the dotted line has been widely reported and accepted;^32–34^ however, the negative correlation on the right-hand side has been overlooked and has not been thoroughly investigated.

**Figure 1:**
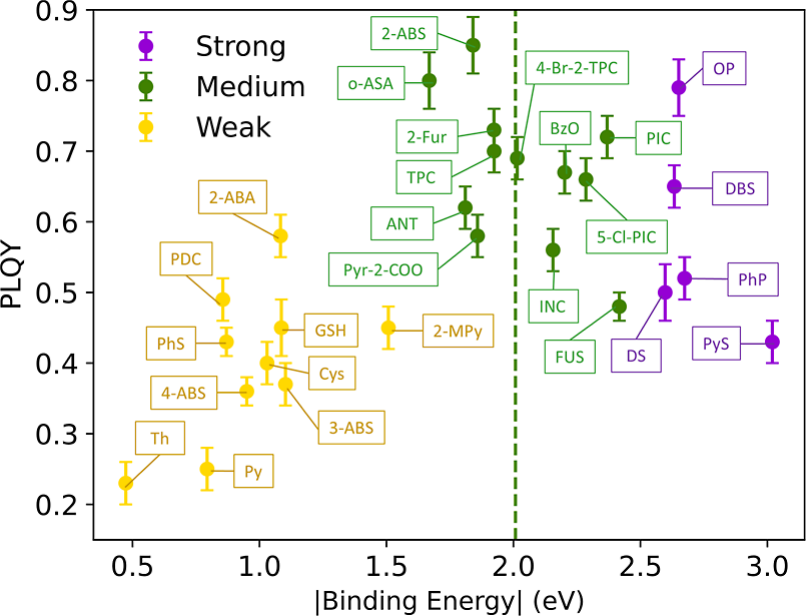
Photoluminescence quantum yield (PLQY) vs binding energy for 27 ligands on the CsPbBr_3_ nanocrystal. The green dotted line represents the binding energy of surface bromide. Purple, strong-binding ligands; green, medium-binding ligands; and yellow, weak-binding ligands.

Based on the calculated binding energy and functional group, the ligands can be classified into three groups: strong-binding, medium-binding, and weak-binding ligands. As shown in Figure 2, ligands in the strong binding category are phosphonate and sulfonate-based anionic ligands, which agrees well with several previous studies reporting their high affinities with undercoordinated lead in halide perovskite nanocrystals.^35–38^ For medium-binding ligands, carboxylate is a dominant functional group. This category contains N-, O-, and S-substituted heterocyclic compounds, such as pyrrole, furan, thiophene, and pyridine, as well as ortho-substituted zwitterionic ligands. Weak ligands consist of thiolate-based anionic ligands, para- and meta-substituted zwitterionic ligands, and neutral molecules (e.g., with the carboxyl group).

**Figure 2:**
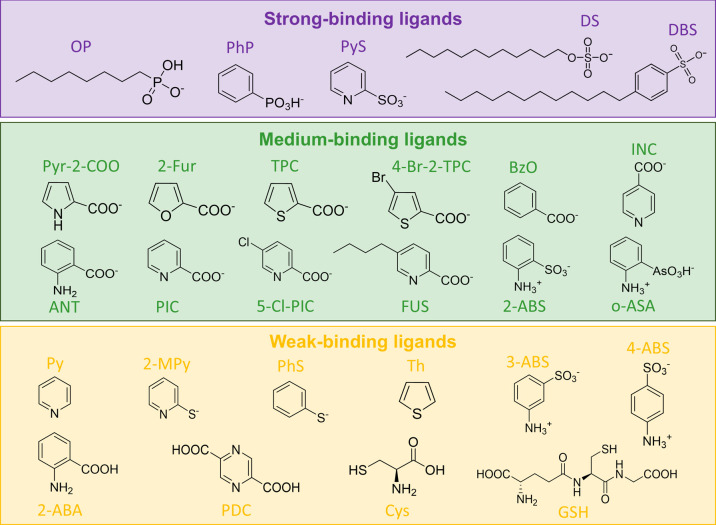
Summary of 27 ligands explored for the surface passivation of cesium lead halide perovskite nanocrystals. The categorization into strong-, medium-, and weak-binding ligands is largely dependent on specific functional groups and their modes of interaction with the halide vacancy.

### 2.2 Binding Structure of the Ligands on the CsPbBr_3_ Nanocrystal

Figure 3 shows the optimized atomic structures of representative ligands in each category. As one can see, for the strong-binding ligands such as phenylphosphonate (PhP) and dodecyl benzenesulfonate (DBS), the oxygen from the phosphonate (−HPO_3_^–^) and sulfonate (−SO_3_^–^) groups forms a monodentate interaction with the uncoordinated lead (Figure 3a). As for the medium-binding ligands, including pyrrole-2-carboxylate (Pyr-2-COO), 2-furoate (2-Fur), 2-thiophenecarboxylate (TPC), 4-bromothiophene-2-carboxylate (4-Br-2-TPC), benzoate (BzO), isonicotinate (INC), and anthranilate (ANT), two oxygens from the carboxylate (−COO^–^) group are bound to the uncoordinated lead (Figure 3b). Unlike the chelating coordination observed for the –COO^–^ group, the carboxyl group (−COOH) of the weak-binding ligands, including anthranilic acid (2-ABA), 2,5-pyrazinedicarboxylic acid (PDC), l-cysteine (Cys), and glutathione (GSH), exhibits monodentate binding via a single oxygen (Figure 3c). Other ligands, such as pyridine (Py) and thiophene (Th) in the weak-binding category, also form monodentate binding via the heteroatom in the aromatic ring.

**Figure 3:**
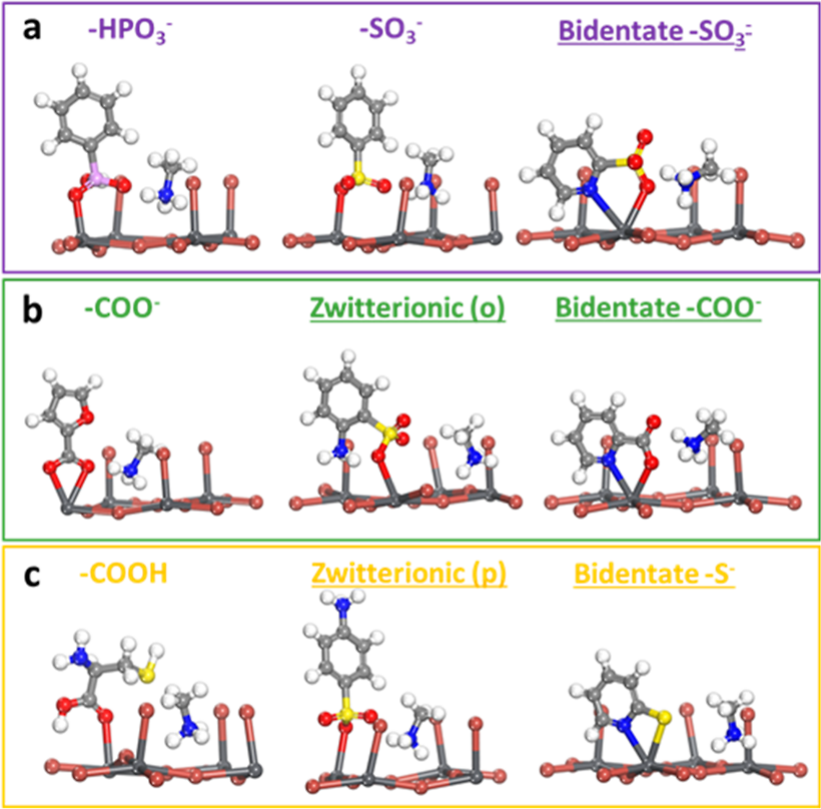
Optimized atomic structures of the strong (top; purple), medium (middle; green), and weak (bottom; yellow) binding ligands on the CsPbBr_3_ nanocrystal. H, white; C, light gray; N, blue; O, red; P, pink; S, yellow; Br, brown; Pb, dark gray.

Besides revealing the binding structure of the ligands at the atomic level, we also identified several interesting trends. First, we found that 2-ABS (ortho), 3-ABS (meta), and 4-ABS (para) ligands have the same monodentate coordination but show decreasing binding energies of −1.84, −1.10, and −0.95 eV, respectively. This can be attributed to the decrease in the hydrogen bonding between the –NH_3_^+^ group and the surface bromides, as the distance between the two functional groups increases.^39,40^ Second, we found that chelate effects can systematically increase the binding energies across all three ligand categories. For example, the strong-binding ligand 2-pyridinesulfonate (PyS) exhibits chelating coordination, where one oxygen from the –SO_3_^–^ group and one nitrogen from the adjacent pyridine ring are bound to the undercoordinated lead. As a result, its binding energy (−3.02 eV) is much stronger than the monodentate dodecyl benzenesulfonate (DBS) with the same sulfonate binding group (−2.63 eV). A similar chelating coordination was also observed and experimentally confirmed^12^ for medium-binding ligands: picolinate (PIC), 5-chloropyridine-2-carboxylate (5-Cl-PIC), and fusarate (FUS), where the oxygen from the –COO^–^ group and the nitrogen from the pyridine ring are involved in the coordination. Thus, these ligands possess more negative binding energies (∼−2.40 eV) than other medium-binding ligands with the –COO^–^ group only. Likewise, the bidentate 2-mercaptopyridine (2-MPy) in the weak-binding ligand category has a stronger binding (−1.51 eV) compared to that of its monodentate counterpart thiophenolate (PhS, −0.87 eV). These observations agree well with previous reports on the stabilization effect of multidentate ligands.^21–23^

Our previous experimental studies have illustrated interactions of several ligands with the PNC surface:^12,39^ in ^1^H NMR, we observed significant broadening of the ^1^H resonance signals of the chelating ligand, PIC, and the zwitterionic ligand, 2-ABS. The broadening of ^1^H resonance signals indicates the proximity of ligands to the PNC surface. In the present study, we found evidence that even ligands with relatively poor passivation performance are associated with the PNC surface (Figure 4). For instance, aniline-4-sulfonic acid (4-ABS) introduced through the solid-state exchange method shows a prominent broadening of ^1^H NMR bands, especially for the proton close to the sulfonate group, indicating sulfonate as the binding group for 4-ABS. For the chelating ligand, PyS, the broadening effect was more prominent for ^1^H­(1) and ^1^H­(2). This broadening pattern is identical to that of PIC, implying a similar affinity of the PNC surface for the nitrogen atom in the pyridine ring. Finally, we must note that due to the competitive adsorption from original synthesis ligands, none of these ligands should be regarded as anchored on the PNC surface but rather as rapidly on–off. They exchange between the surface and the solution phase and reach dynamic equilibria with the native ligands. We, therefore, increased the concentration of passivation ligands to the level that no more passivation effect could be observed (Figure S1). This passivation strategy, together with the clear observation of the surface association for different ligands with NMR, suggests their different passivation capabilities are not due to the surface coverage but to their electronic effects, which will be discussed in the next section.

**Figure 4:**
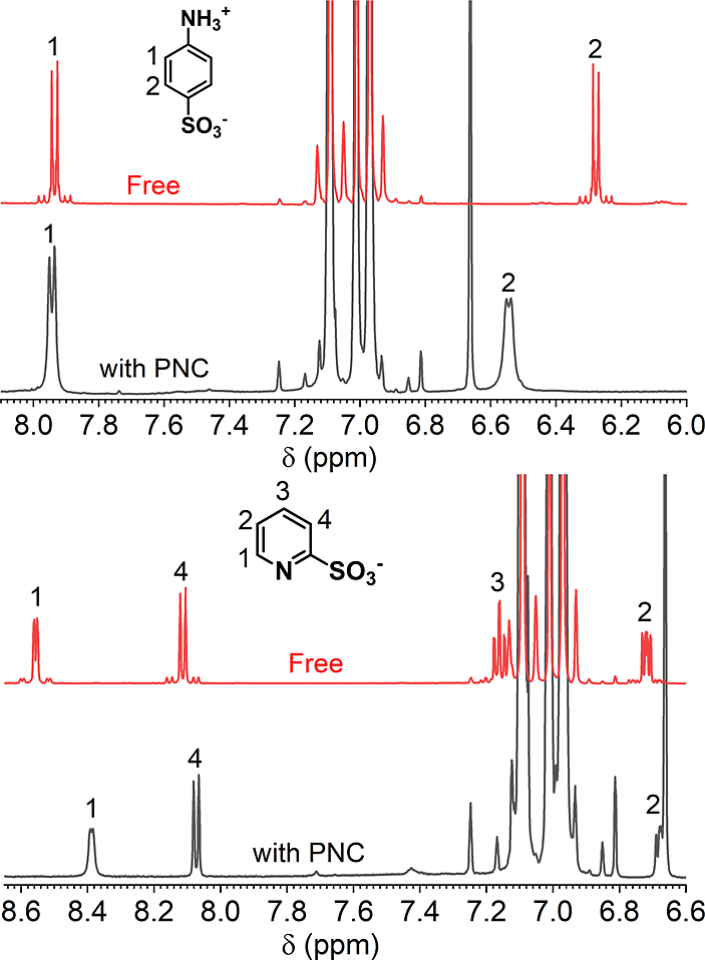
^1^H NMR of 4-ABS and PyS ligands with perovskite nanocrystals (black) in toluene-*D*_8_ at their best passivation points, when the PLQYs of the samples reached their maxima for specific ligands, as shown in the Supporting Information. Specifically, the 4-ABS exchange was carried out for 24 h to saturate the system, reaching a PLQY of 0.36±0.02, and for PyS, 1500 e.q. (255 μM) of the ligand was added to achieve a PLQY of 0.5. For 4-ABS, spectra of the free 4-ABS (solvated by OA and OlAm) and PyS/OlAm (red) in toluene-D_8_ are provided for comparison.

### 2.3 Fundamental Understanding of the Detrimental Effects of Strong-Binding Ligands

To understand the counterintuitive decrease in PLQY for strong-binding ligands, we first established an electronic baseline. As shown in Figure S3, we calculated the density of states (DOS) for defect-free perovskite nanocrystals and nanocrystals containing only a halide vacancy for CsPbCl_3_, CsPbBr_3_, and CsPbI_3_. The defect-free systems exhibit a clean bandgap with the Fermi level located at the valence band maximum, consistent with charge-balanced semiconducting behavior. Our calculated bandgap agrees well with previous studies that employed the Perdew, Burke, and Ernzerhof (PBE) functional.^41,42^ In contrast, introducing a halide vacancy shifts the Fermi level toward (or into) the conduction band minimum (CBM), reflecting the donor-like character of the vacancy and the emergence of defect-associated states. We further computed DOS plots for all 27 ligands in the three systems (Figures S4–S6). Most weak-binding ligands do not electronically passivate the halide vacancy; the Fermi level remains near or within the CBM, indicating ineffective defect passivation. The differences in Fermi level positions between medium- and strong-binding ligands are not pronounced.

To further distinguish these categories, we performed Bader charge analysis.^43^ We find that the stronger electron-withdrawing nature of sulfonate and phosphonate groups in strongly bound ligands leads to greater electron transfer from the perovskite surface, inducing hole states at the ligand–perovskite interface. These interfacial hole states are detrimental to the PLQY because they promote nonradiative recombination. We plotted the Bader charges of the ligands against their binding energies (Figure 5). The resulting linear relationship confirms that stronger binding arises from increased ionic (charge-transfer) interaction. This trend indicates that strong-binding ligands possess larger Bader charges, leading to a greater electron withdrawal and a higher density of interfacial hole states.

**Figure 5:**
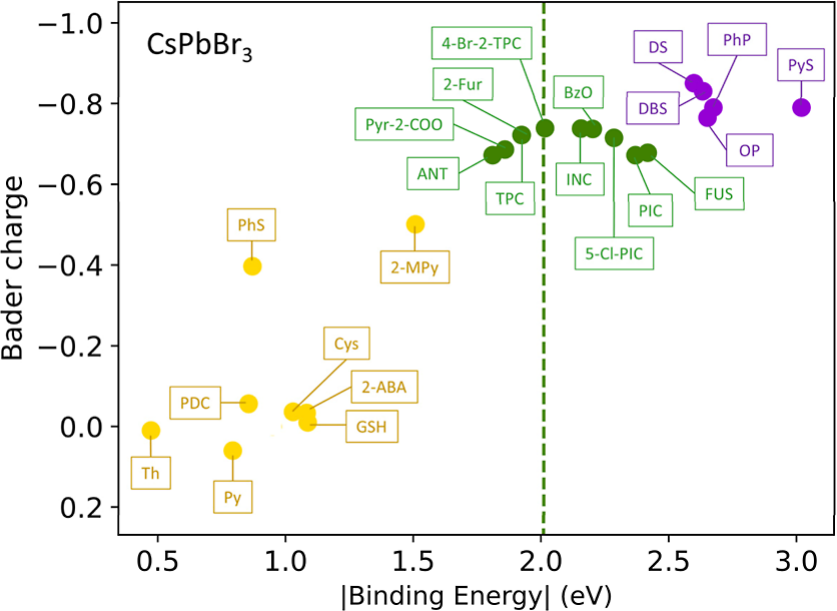
Ligand Bader charge vs binding energy at the CsPbBr_3_ surface. The dotted line shows the binding energy of bromide on the pristine, defect-free surface. The reported linear trend is evaluated using the nonzwitterionic subset of ligands only.

To illustrate the influence of the induced trap, we compared the exciton relaxation dynamics of CsPbBr_3_ perovskite nanocrystal samples passivated by PIC and PyS, using time-resolved photoluminescence (TR-PL) and transient absorption (TA) spectroscopy (Figure 6). The excellent passivation effect of PIC, which has an appropriate binding energy and a moderate Bader charge, was demonstrated by the diminishing of the initial fast exciton decay component caused by deep traps related to surface defects and the elongation of the overall exciton lifetime to 4.84 ± 0.04 ns, as indicated by the decay kinetics of TR-PL. The ground state bleach (GSB) signal of TA of the PIC passivated perovskite nanocrystal can be fit with a double-exponential decay, with time constants of 150 ps and 4.9 ns, respectively (Table 1). The former corresponds to trapping at intrinsic defects that cannot be completely passivated by the surface ligands, while the latter agrees with the exciton relaxation rate captured by TR-PL.

**Figure 6:**
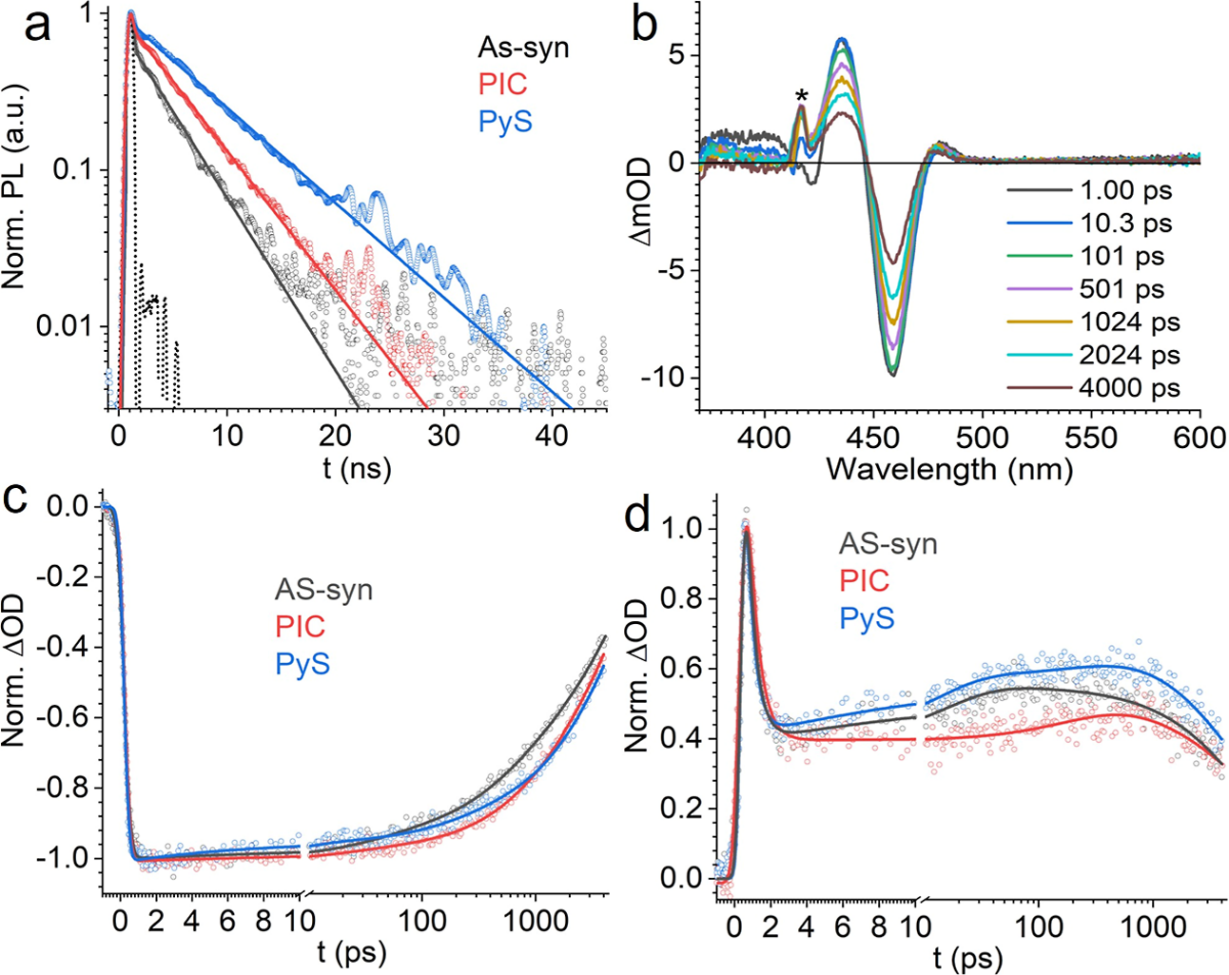
Comparison of exciton relaxation kinetics for as-synthesized, PIC-passivated, and PyS-passivated perovskite nanocrystal samples. (a) TR-PL kinetics with 420 nm excitation. The dotted line indicates the IRF signal from directly measuring the pump scattering. (b) Selected TA spectra of the PyS-passivated perovskite nanocrystal samples at different time delays. “*” denotes the spectral region affected by the pump scattering. (c,d) TA kinetic traces extracted for the GSB signals at 457 nm and the photoinduced absorption signals at 477 nm. Kinetic fitting parameters are listed in Table 1. The PLQY of the as-synthesized PNC used in the time-resolved spectroscopic study is 0.33 ± 0.02, slightly higher than the steady state measurements. However, the PLQYs of the PIC- and PyS-passivated sample still fall in the same range as those reported in Table S1.

**Table 1:** Kinetic Fitting Parameters of TR-PL and TA Data for as-Synthesized PIC- and PyS-Passivated Samples

		TA			
samples	τ_TRPL_ (ns)^a^	signal (nm)	τ_1_ (ps) A_1_ (%)	τ_2_ (ps) A_2_ (%)	τ_3_ (ns) A_3_ (%)
as-syn	3.94 ± 0.03	GSB	37 ± 9	520 ± 80	5.6 ± 0.3
		457	5.7 ± 0.9	17 ± 2	76 ± 2
		PIA^b^	16 ± 3		6.3 ± 0.9^c^
decay		477	growth		
PIC	4.84 ± 0.04	GSB		150 ± 20	4.9 ± 0.1
		457		6.3 ± 0.5	94 ± 1
		PIA^b^		220 ± 70	4.6 ± 0.5^c^
470				growth	decay
PyS	7.08 ± 0.03	GSB	6.5 ± 0.9	190 ± 40	5.9 ± 0.1
		457	5.1 ± 0.5	6.3 ± 0.5	89 ± 1
		PIA^b^	12 ± 2	260 ± 90	5.3 ± 0.7^c^
decay		470	growth	growth	

a An ultrafast decay component with time constants of ∼0.03 ns is included to fit the early kinetics, as shown in Table S2, but was not reported because it is much faster than the instrumental response (0.57 ns) of the system.

b The initial rapid decay component with a time constant of ∼0.6 ps was not listed.

c Amplitudes with infinite long time-constants were included to fit the slow decay process.

PyS, with greater binding energy and higher Bader charge, demonstrates a lower PLQY than PIC. However, according to the TR-PL measurement, PyS-passivation does not shorten the observed exciton lifetime as usually expected in poorly passivated samples but instead elongates the lifetime to 7.08 ± 0.03 ns. This lifetime is even longer than most reported CsPbBr_3_ perovskite nanocrystals with close-to-unity quantum yields,^39,44^ and cannot be attributed to the dielectric shielding effect, due to the similar dielectric properties of PyS and PIC.^45^ TA kinetics captured exciton trapping in the PyS-passivated sample as an ultrafast GSB decay with a time constant of 6.5 ± 0.9 ps. We also noticed that the PyS-passivated sample has the strongest growth of the photoinduced absorption (PIA) band on the lower energy side of the GSB signal among the three test samples. This PIA signal is related to the Stark effect induced by separated charge carriers at the interface.^46,47^ The prominent rise of this PIA signal synchronizes with the initial fast decay of the GSB signal in the PyS-passivated sample, suggesting that the exciton decay is likely caused by surface carrier trapping induced by the PyS ligand. In contrast, the PIC-passivated sample shows the smallest growth amplitude, implying that the adsorption of PIC eliminates carrier trapping on the surface. This extra trapping induced by the surface-attached PyS ligand causes the lowering of the PLQY. On the other hand, the trapping process results in localized holes, which impede the carrier recombination as observed by the elongated TR-PL lifetime (7.08 ± 0.03 ns) and the slowdown of GSB decay (5.9 ± 0.1 ns) in TA.

### 2.4 Design Principles for High PLQY and Experimental Validations

Based on the above discussions, we propose the following design principle: To obtain halide perovskite nanocrystals with high PLQY, ligands with binding energies close to those of surface halide species should be selected to achieve sufficiently strong ligand-perovskite interactions while preventing the formation of hole states above the Fermi level. We further extended our study from CsPbBr_3_ to CsPbCl_3_ and CsPbI_3_ nanocrystals. As shown in Figure 7, the purple and yellow dotted lines were plotted in a similar manner to represent the binding energy of the surface chloride (−2.34 eV) and iodide (−1.55 eV), respectively. We observe that for CsPbCl_3_, the dotted line is brought closer toward the strong- and medium-binding ligands, whereas for CsPbI_3_, it is considerably closer toward the weak-binding ligands. This trend agrees nicely with our previous observation for CsPbBr_3_ favoring medium-binding ligands, since an increasing binding energy between the halide and the perovskite surface is expected in the order of iodide, bromide, and chloride, corresponding to the decreasing atomic radius. We observed an overlap between the medium- and strong-binding categories in the case of CsPbCl_3_, with several medium-binding ligands having similar binding energies compared to those of chloride. Based on this, we provide design principles that both strong- and medium-binding ligands shown in Figure 2 can be good candidates for chloride vacancy passivation of CsPbCl_3_, whereas weak-binding ligands would be favored for iodide vacancy passivation of CsPbI_3_ nanocrystals in the quantum-confined regime.

**Figure 7:**
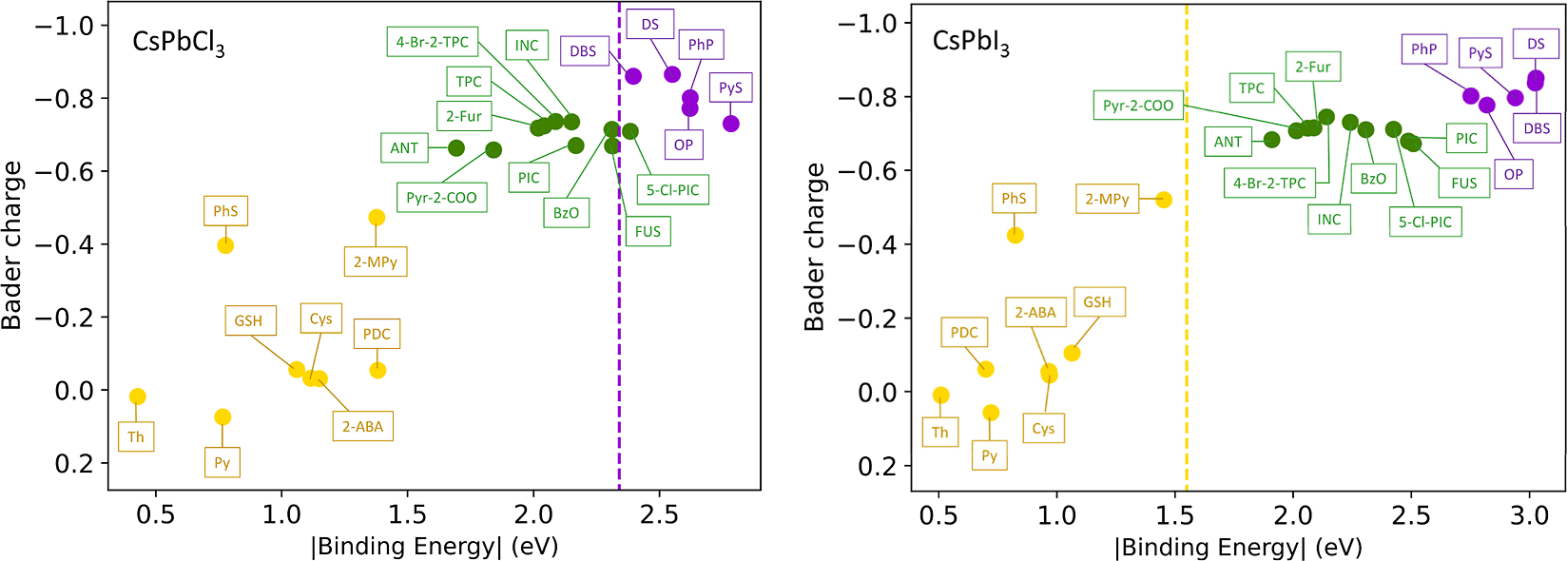
Ligand Bader charge vs binding energy at the CsPbCl_3_ (left) and CsPbI_3_ (right) surfaces. The dotted lines show the binding energy of chloride and iodide on the pristine, defect-free surface, respectively. The reported linear trend is evaluated using the nonzwitterionic subset of ligands only.

We performed passivation experiments on CsPbCl_3_ and CsPbI_3_ nanocrystals to validate our computational predictions. Three representative passivation ligands from different binding energy categories were selected, and the results are listed in Figure 8. For CsPbCl_3_, the PL intensity increases with the binding energy of the passivation ligand, with strong-binding ligands, such as OP, providing the most effective passivation. Notably, OP enhanced PLQY by almost 3-fold, reaching 0.042 ± 0.02. PIC, a medium-binding ligand and one of the most effective passivators for CsPbBr_3_, showed only moderate passivation (blue curve) for CsPbCl_3_. The CsPbCl_3_ perovskite nanocrystals tested here exhibited a first-excitonic band maximum at 383 nm and an emission peak at 391 nm, bluer than most reported samples (∼400 nm emission), indicating a stronger susceptibility to deep traps. Despite their large binding energies, PyS and DBS did not achieve the predicted passivation, likely because their bulky aromatic sulfonate groups did not fit well with the small CsPbCl_3_ lattice. This result suggests that lattice size can be another important factor when selecting passivation ligand structures.^21^ Weak-binding ligands such as 3-ABS and 2-MPy did not improve the PLQY; in fact, 3-ABS decreased the PL, likely due to its zwitterionic nature, enabling it to pair with native ligands and strip them from the surface into the solution. Overall, these results confirm that strong- or medium-binding ligands can serve as chloride-equivalents to effectively passivate halide vacancies on the CsPbCl_3_ surface.

**Figure 8:**
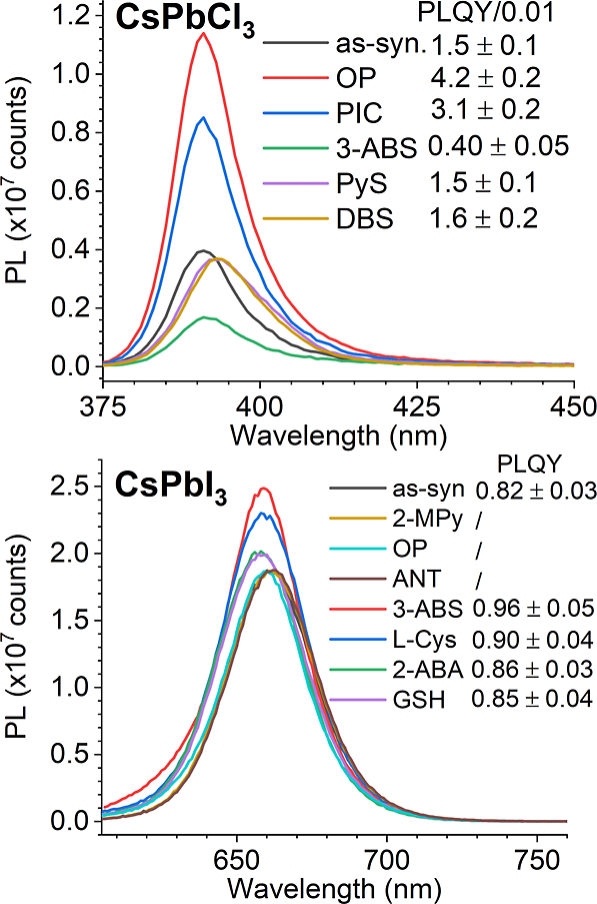
Passivation effects of ligands with different binding energies for CsPbCl_3_ and CsPbI_3_ nanocrystals in toluene. The absolute quantum yield of each sample is labeled in the legend. PLQYs of CsPbCl_3_ samples in the legend were divided by 0.01.

For CsPbI_3_, which is known for its defect tolerance, the as-synthesized sample already exhibited a high initial PLQY of 0.82 ± 0.04. Passivation with the selected ligands showed the opposite trend to CsPbCl_3_, as predicted: weak-binding ligands with low Bader charge values, such as Cys, GSH, 3-ABS, and 2-ABA, further enhanced the PLQY. In particular, 3-ABS passivation yielded a PLQY approaching unity. Notably, Cys has previously been used as a passivation ligand to improve the performance of red-light LEDs based on CsPbI_3_ nanocrystals.^48^ In contrast, ligands with high Bader charges, such as 2-MPy, OP, and ANT, produced little improvement in PL intensity even when their binding energies were close to that of iodide. However, this does not imply that such ligands cannot be beneficial for stabilizing the surface chemistry or phase of CsPbI_3_. For example, 2-MPy has been reported to improve stability and device performance in CsPbI_3_-based applications.^49^ The comparison between 2-ABA and ANT highlights the impact of the ligand charge state on passivation performance. The neutral 2-ABA demonstrated effective passivation, whereas its closely related, negatively charged analogue ANT, bearing a stronger electron-withdrawing group, did not. Based on these results, we predict that the most promising candidates for passivating iodide vacancies in CsPbI_3_ fall within the category of weak-binding ligands with low Bader charges.

Beyond rationalizing the behavior of the 27 ligands examined here, the binding-energy descriptor established in this work provides a predictive framework for the design of new passivation ligands. Specifically, effective ligands should possess anchoring groups (as shown in Figure 2) whose binding energies closely match that of the native halide species for a given composition, thereby ensuring sufficient defect passivation without inducing excessive charge transfer and interfacial hole states. In addition to headgroup identity and charge, denticity and steric compatibility with the perovskite lattice must be considered, as multidentate coordination can enhance stability while bulky groups may limit surface accessibility. Taken together, these chemically intuitive parameters enable qualitative prediction of passivation efficacy and expected PLQY trends across different halide compositions.

It is important to note that our analysis does not take into account intrinsic defects that cannot be directly passivated through surface treatments,^12,50^ nor the influence of surface ligands on the phase transformation of CsPbI_3_ nanocrystals, which can also substantially affect the PLQY.^51,52^ In addition, in solution, the effective binding affinity of ligands to the perovskite surface also depends on their interaction with the surrounding solvents. As a result, some ligands that most effectively enhance the PLQY may require excess quantities to achieve full passivation. Likewise, due to solvation effects, certain long-chain ligands with strong solvation behavior (e.g., OP) may exhibit slight deviations from the general trend. Here, we aim to employ a simplified model that correlates the experimentally measured PLQY with the DFT-calculated ligand binding energy, thereby emphasizing the dominant role of ligand-perovskite interactions and establishing that an appropriate “halide-equivalent” binding energy serves as a guiding principle for identifying effective passivation ligands.

## 3 Conclusions

A systematic study to understand and design ligands for enhanced PLQY in cesium lead halide perovskite nanocrystals was achieved. Through combined first-principles DFT calculations and experiments, a volcano relationship between the ligand binding energy and PLQY in quantum-confined CsPbBr_3_ nanocrystals was identified. This nonmonotonic relationship arises because ligand binding energy plays two competing roles: (1) it must be sufficiently strong to passivate surface halide vacancies, but (2) if binding becomes overly strong, the ligand withdraws more electron density (i.e., higher Bader charge) and induces hole states at the interface. Time-resolved spectroscopy directly shows that strong-binding PyS-passivated CsPbBr3 exhibits ultrafast trapping and strong photoinduced absorption growth consistent with interfacial charge separation. We demonstrated that ligands with binding energies close to that of the surface halide species offer the best passivation by balancing the two. Our proposed design principle can be extended to quantum-confined CsPbCl_3_ and CsPbI_3_ nanocrystals, as validated by experiments. This work provides valuable guidelines for screening effective surface ligands based on a simplified descriptor. With this, we hope to pave the way for the high-throughput computational screening of surface ligands to ultimately contribute to the development of more stable, high-performance optoelectronic devices based on halide perovskite nanocrystals.

## 4 Methods

### 4.1 DFT Calculations

DFT calculations were performed using the Vienna Ab initio Simulations Package (VASP).^53^ The ion–electron interaction was described by the projector-augmented wave method,^54^ with a planewave cutoff energy set to 400 eV. The exchange–correlation interaction was described by the generalized gradient approximation method of PBE.^55^ For structural relaxations, the PBE functional was employed with a 3 × 3 × 1 Monkhorst–Pack *k*-point mesh. The self-consistent calculations for the total DOS were performed on the relaxed structures using the PBE functional with an 11 × 11 × 1 *k*-point mesh. The energy convergence criterion of 1 × 10^–4^ eV and force criterion of 0.025 eV/Å were applied throughout the calculations. The van der Waals correction was included using the DFT-D3 method of Grimme with a zero-damping function. A slab of cubic CsPbX_3_ (100) with a CsX termination was modeled with seven atomic layers of a 2 × 2 supercell, separated by a 15 Å vacuum along the *z*-direction. The topmost layer contained a cesium vacancy with an adsorbed methylammonium and a halide vacancy with an adsorbed ligand of interest. During structural relaxations, the top three atomic layers of the CsPbX_3_ and the surface-bound ligands were allowed to relax, while the bottom four layers were fixated. The binding energy of the ligands (Δ*E*) was computed as

$$\Delta E=E_{total}-\left(E_{vacancy}+E_{ligand}\right)$$

where *E*_total_, *E*_vacancy_, and *E*_ligand_ represent the energies of the slab with adsorbed ligands (i.e., vacancies-passivated slab), the slab with a halide vacancy, and the isolated ligand, respectively. A negative Δ*E* suggests favorable spontaneous adsorption, while a greater magnitude indicates stronger binding.

### 4.2 Experiments

#### 4.2.1 Preparation of CsPbX_3_ Nanocrystals

Perovskite nanocrystals were synthesized using a method adapted from the work of Son and coauthors,^30^ and more details can be found in our previous report.^12^ Briefly, a cesium oleate (CsOA) precursor was prepared by dissolving 250 mg of CsCO_3_ in a solution consisting of 0.9 mL of OA and 9 mL of ODE at 120 °C under vacuum. The CsOA precursor was then heated to 150 °C under N_2_. In a separate flask, 0.4 mM PbX_2_, 1.6 mM anhydrous ZnX_2_, and 10 mL of ODE were degassed under vacuum at 90 °C for 1 h. The halide salts were then fully dissolved by adding a 9 mL 1/1 (v/v) mixture of OA and OlAm while maintaining the temperature at 90 °C. 0.8 mL of CsOA precursor solution was swiftly injected into the Pb precursor solution under rigorous stirring, and the reaction took 60 s before cooling down to room temperature with an ice bath. The reaction mixture was then centrifuged at 3500 rpm for 30 min to remove the precipitate. The upper solution was aged for a few hours and centrifuged again to remove the residual salts. Acetone was added dropwise to the clear solution to precipitate the perovskite nanocrystal of the required sizes. We chose CsPbBr_3_ nanocrystals with a bandgap absorption of 458 nm for the present study. CsPbCl_3_ and CsPbI_3_ nanocrystals used in the present study have bandgap absorption at peaks 382 and 656 nm, respectively.

#### 4.2.2 Time-Resolved Spectroscopy

Time-resolved photoluminescence (TR-PL) and subpicosecond TA experiments were carried out using homemade setups powered by a pulse-regenerated amplifier (Regen, Phidia-C, Uptek), outputting 1 kHz, 800 nm pulses with a 150 fs pulse length. More detailed descriptions of the setups have been published previously. For both experiments, we excited the perovskite nanocrystal samples at 420 nm. The pump pulse was produced via the fourth-harmonic generation of the 1680 nm idler pulse output from an optical parametric amplifier (TOPAS, Light-Conversion).

In the TR-PL experiment, photoluminescence of the sample stimulated by a 420 nm pump pulse was directly collected by a photodiode detector (DET025AL, Thorlabs). An oscilloscope (TDS 680C, Tektronix) synchronized with the laser output was employed to measure the photocurrent from the photodiode. The time resolution of TR-PL was ∼570 ps according to the measurement of the width of a pump scattering signal. The TR-PL kinetic traces were fit to a convoluted function of the IRS function (fwhm = 0.57 ns) and a double-exponential decay, as illustrated in Figure 6A. The exact fitting parameters were listed in Table S2.

The TA experiment employed a 1:1 (v/v) H_2_O/D_2_O mixture to generate a white light supercontinuum between 430 and 700 nm as the probe pulse. The probe beam was divided into two arms: one overlapped with the 420 nm pump beam at the sample to probe the TA signal and the other was employed as the reference beam to cancel the pulse-to-pulse fluctuation. The two arms were diverged vertically by about 2.4 cm at their focal points in the sample plane and then collimated and focused onto the slit of the spectrometer (SpectraPro-300i, Acton). Their signal and reference WL were dispersed in two vertically aligned photodiode arrays (EB Stressing) by a 300 groove/mm grating. TA signals were calculated using the equation, $\Delta OD=-\log\left({\left(\frac{I_{S}}{I_{R}}\right)}_{pump‐on}/{\left(\frac{I_{S}}{I_{R}}\right)}_{pump‐off}\right)$ . The TA Instrumental response is ∼300 fs, as determined from the solvent’s stimulated Raman signal.

## Supplementary Material



## Notes

Further details on the atomistic model, materials, and passivation tests, a summary table of ligand binding energies and experimental PLQY, PL spectra for all CsPbBr_3_ samples, and DOS plots for all CsPbX_3_ systems (PDF)
