# Supplementary material for: Design Principles for Surface-Passivating Ligands of Cesium Lead Halide Perovskite Nanocrystals in the Strongly Quantum-Confined Regime
Source: Chem Mater. 2026 Mar 19;38(7):3429–38. doi: 10.1021/acs.chemmater.5c03187 (PMC13084979; doi:10.1021/acs.chemmater.5c03187)
Supplement: Supplementary file 1 [file cm5c03187_si_001.pdf]

Online Supporting Information for

Design Principles for Surface-Passivating Ligands of Cesium Lead Halide Perovskite Nanocrystals in the Strongly Quantum-Confined Regime

Seungjun Cha,[1,#] Courtney Brea[2,3,#], Aaron Malinoski[2,3, #], Chen Wang[2,3,*], Guoxiang Hu[1,4,*]

[1] School of Materials Science and Engineering, Georgia Institute of Technology, Atlanta, GA 30332, USA

[2] Department of Chemistry and Biochemistry, Queens College, City University of New York, New York, NY 11367, USA

[3] The Graduate Center, City University of New York, New York, NY 10016, USA

[4] School of Chemistry and Biochemistry, Georgia Institute of Technology, Atlanta, GA 30332, USA

[#] S.C., C.B., and A.M. contributed equally

*Corresponding author.

Email: chen.wang@qc.cuny.edu, emma.hu@mse.gatech.edu

METHODS

1. Additional computational details

The defect model used in this study is based on the CsX-terminated perovskite nanocrystal surface, as suggested by previous computational and experimental studies.[1,2] We focused on L-type passivation ligands targeting halide vacancies on the perovskite nanocrystal surface, since these are the dominant surface defects that generate interband states and are responsible for carrier trapping.[3,4] In addition to halide vacancies, Cs^+ vacancies are also common and some of these vacancies are passivated by the ammonium cations.[1,2] Several experimental studies, including our own previous work,[5] have shown that anionic ligands coordinated at halide vacancies are often paired with ammonium cations on the perovskite nanocrystal surface. Both Cs^+ vacancies and adsorbed ammonium are generally reported to introduce shallow states near the band edges rather than deep mid-gap trap states.[3,6] They are therefore not considered dominant nonradiative recombination centers and do not significantly perturb the electronic structure within the bandgap.

In our passivation experiments, L-type ligands were generated by deprotonating their acidic groups with oleylamine, or, in the case of bidentate zwitterionic ligands, via adjacent amine groups. In both cases, the nearby ammonium cations could enhance ligand binding affinity[7–10], but their direct passivation effect was negligible compared to that of the L-type ligands, as shown in Figure S2. Accordingly, in our computational model of the passivated perovskite nanocrystal surface, the surface halide was replaced by an L-type ligand, while either a methylammonium cation or the ammonium group of a zwitterionic ligand substituted the nearby Cs^+ site. For each ligand, we examined multiple plausible coordination modes at the halide vacancy site, and the energetically most favorable configuration was reported. The binding configurations of representative ligands on perovskite nanocrystal surfaces are illustrated in Figure 3.

2. Additional experimental details

Materials. Unless specified, the following chemicals were used as received without any further treatment. Hexanes (Certified ACS, Fisher). Toluene (Certified ACS, Fisher). The abovementioned solvents were dried with the molecular sieve 4Å overnight before being used. Acetone (Certified ACS, Fisher). 1-octadecene (ODE, 90%, Acros). Oleic acid (OA, 90%, Sigma-Aldrich). Oleylamine (OlAm, 70%, Sigma-Aldrich). Lead (II) chloride ($PbCl_2$, 99.999%, Alfa Aesar). Lead (II) bromide ($PbBr_2$, 99.998%, Alfa Aesar). Lead (II) iodide (PbI_2, 99.9985%, Alfa Aesar). Anhydrous zinc chloride ($ZnCl_2$, ACS grade, Alfa Aesar). Anhydrous zinc bromide ($ZnBr_2$, 99.9%, Alfa Aesar). Anhydrous zinc iodide (ZnI_2, 99.9%, Alfa Aesar). Cesium carbonate (Cs_2CO_3, 99.9%, Acros). Benzylic acid (99.5%, Alfa Aesar). Picolinic acid (99%, Acros). 2-thiophenecarboxylic acid (99%, Acros). 2-pyrrolecarboxylic acid (99%, Alfa Aesar). 2-furancarboxylic acid (98%, Acros). Isonicotinic acid (99%, Acros). 9,10-diphenylanthracene (99%, Alfa Aesar). Phenylphosphonate (97%, Thermo Scientific Chemicals). Dodecyl sulfate (sodium salt, 99%, Thermo Scientific Chemicals). Benzenesulfonic acid (anhydrous, >98%, TCI). Pyridine (certified ACS, Fisher). Thiophene (>98%, TCI). 2-Aminobenzenesulfonic acid (>99%, TCI). 2-3-Aminobenzenesulfonic acid (>99%, TCI). 4-Aminobenzenesulfonic acid (>99%, TCI). o-Arsanilic acid (98%, Thermo Scientific). 2-Pyridinesulfonic acid (97%, Thermo Scientific). (Thiophenol, 99%, Acros). 2-Mercaptopyridine (98%, Thermo Scientific). Anthranilic acid (>99%, Sigma-Aldrich). 5-Chloropyridine-2-carboxylic acid (95%, Thermo Scientific). Fusaric acid (99%, Thermo Scientific). 4-decyl benzenesulfonic acid (95%, with isomers, Thermo Scientific). Pyrazine-2,5-dicarboxylic acid (95%, Thermo Scientific). L-Cysteine (>98%, Thermo Scientific). L-Glutathione reduced form (>97.0%, TCI).

Passivation tests. The chemical structures of the 27 passivation ligands tested in the present study are summarized in Figure 2 and grouped according to their binding energy to the perovskite surface. All the ligands from chemical suppliers are commercially available and affordable. The passivation effects of various ligands on $CsPbBr_3$ nanocrystals were determined by comparing the photoluminescence quantum yield (PLQY) of the samples after applying the ligands. All steady-state PL measurements were carried out using a Horiba FluoroMax 3 fluorometer with an excitation wavelength of 405 nm. The $CsPbBr_3$ nanocrystal samples were dissolved in toluene, and the optical density of the solution at 405 nm was adjusted to 0.2 cm^{-1}. Absolute PLQYs of $CsPbBr_3$ and $CsPbCl_3$ nanocrystal samples were determined using a 9,10-diphenyl anthracene (DPA) solution as the reference. PLQYs of $CsPbI_3$ perovskite nanocrystal samples were measured using cresyl violet as the standard. The excitation wavelengths of PL measurements were chosen as 350 nm, 405 nm, and 580 nm for $CsPbCl_3$, $CsPbBr_3$, and $CsPbI_3$, respectively, and the optical densities of the perovskite nanocrystal samples, and the fluorescence reference standards were adjusted to be the same, 0.2 cm^{-1}, at the excitation wavelengths.

The passivation ligands were applied in two ways, depending on their solubility in toluene. For ligands with poor solubility, such as 2-ABS, 3-ABS, 4-ABS, o-ASA, PDC, Cys, and GSH, we directly added the solid-state compounds into a 2 μM toluene solution of as-synthesized nanocrystals. The mixtures were stirred overnight so the solid ligands could be slowly brought into solution by pairing them with the oleate and oleylammonium ligands in the as-synthesized sample.[10] Undissolved solids were removed by centrifugation. The nanocrystal solutions were then filtered with syringe filters with a pore size ≤0.45 μm and diluted to the needed concentration for PL measurements.

All other ligands were introduced to the nanocrystal solutions with a titration approach. Pyridine (Py) and thiophene (Th) were added directly to reach 20,000 e.q. per nanocrystal. Acidic ligands were added to the nanocrystal solutions as their oleylammonium salts, which were prepared by dissolving the acidic ligands with OlAm in a 1-to-1 mole ratio in toluene. We titrated the ligand-OlAm solution to the nanocrystal samples while monitoring their PL intensities. For different ligands, 350 to 2700 e.q. of applied ligands per nanocrystal would be required to achieve the maximum PL intensity. For pyridine and thiophene, pure ligands were added up to 20000 e.q., and we observed only a marginal increase in the PLQY. The maximum volume of the added ligand solution was kept under 3% of the original nanocrystal solutions.

In the solution phase, surface defect passivation is a dynamic process rather than a steady-state adsorption. In our previous work, we demonstrated that two ligands used in the present study, picolinate (PIC) and aniline-2-sulfonic acid (2-ABS), both of which exhibit strong passivation effects, undergo continuous on-off exchange with the surface, reaching dynamic equilibria with the native ligands.[5,10] The distribution of passivation ligands is governed by the binding energy of their anchoring motifs to surface sites, their interactions with solvent molecules, and competitive adsorption from the original synthetic ligands. Relatively stable binding of PIC and 2-ABS was only observed after specialized purification treatments, which, however, reduced the PLQY from its optimal passivation level. In the present study, to compare the passivation capacities of different ligands, we did not aim for steady-state adsorption. Instead, we applied a saturated passivation procedure, adding a sufficient excess of ligand until the PLQY reached its maximum possible value, as shown in Figure S1. For ligands soluble in toluene, we titrated them into pristine perovskite nanocrystal solutions while monitoring PL changes; for different ligands, 350-2700 equivalents were required to reach the plateau in PLQY. For insoluble ligands, we employed a solid-state

exchange procedure, allowing extended contact between the perovskite nanocrystal sample and the ligand until equilibrium distribution was achieved. ^{1}H NMR data for perovskite nanocrystals passivated by 2-pyridinesulfonate (PyS) and aniline-4-sulfonic acid (4-ABS) using the solid-state exchange method are provided in Figure 4. These examples demonstrate that, like PIC and 2-ABS, PyS and 4-ABS also undergo dynamic adsorption-desorption on the perovskite nanocrystal surface. The saturating passivation method employed in this study may not apply to cases of Py and Th, due to their low binding energies and strong interactions with toluene. Ligands with such low binding affinities cannot effectively passivate PNCs in toluene.

Table S1. DFT-calculated binding energy, experimental PLQY in toluene, and equivalence added to reach the maximum PL for the 27 ligands studied on $CsPbBr_3$ nanocrystals. *Indicates ligands introduced through solid-state exchange.

**Ligands**	**Binding energy**	**PLQY**	**Ligand concenrations** e.q. (μM)
**Octylphosphonate (OP)**	-2.65	0.79±0.04	350 (60)
**Phenylphosphonate(PhP)**	-2.67	0.52±0.03	1100 (187)
**2-Pyridinesulfonate (PyS)**	-3.02	0.43±0.03	1500 (255)
**Dodecyl sulfate (DS)**	-2.60	0.50±0.04	*
**Dodecyl benzenesulfonate (DBS)**	-2.63	0.65±0.03	1000 (170)
**Pyrrole-2-carboxylate (Pyr-2-COO)**	-1.86	0.58±0.03	1500 (255)
**2-Furoate (2-Fur)**	-1.92	0.73±0.03	1000 (170)
**2-Thiophenecarboxylate (TPC)**	-1.92	0.70±0.03	1200 (204)
**4-Bromothiophene-2-carboxylate (4-Br-2-TPC)**	-2.01	0.69±0.03	1600 (272)
**Benzoate (BzO)**	-2.20	0.67±0.03	2700 (459)
**Isonicotinate (INC)**	-2.16	0.56±0.03	1500 (255)
**Anthranilate (ANT)**	-1.81	0.62±0.03	2100 (357)
**Picolinate (PIC)**	-2.37	0.72±0.03	700 (119)
**5-chloropyridine-2-carboxylate (5-Cl-PIC)**	-2.29	0.66±0.03	550 (94)
**Fusarate (FUS)**	-2.42	0.48±0.02	1200 (204)
**2-Aminobenzenesulfonic acid (2-ABS)**	-1.84	0.85±0.04	370 (63)*
**o-Arsanilic acid (o-ASA)**	-1.67	0.80±0.04	*
**Pyridine (Py)**	-0.79	0.25±0.03	20000 (3400)
**2-Mercaptopyridine (2-MPy)**	-1.51	0.45±0.03	500 (85)
**Thiophenolate (PhS)**	-0.87	0.43±0.02	2000 (340)
**Thiophene (Th)**	-0.47	0.23±0.03	20000 (3400)
**3-Aminobenzenesulfonic acid (3-ABS)**	-1.10	0.37±0.03	410(70)*
**4-Aminobenzenesulfonic acid (4-ABS)**	-0.95	0.36±0.02	470 (80)*
**Anthranilic acid (2-ABA)**	-1.08	0.58±0.03	1600 (272)
**2,5-pyrazinedicarboxylic acid (PDC)**	-0.86	0.49±0.03	*
**L-cysteine (Cys)**	-1.03	0.40±0.03	*
**Glutathione (GSH)**	-1.09	0.45±0.04	*

Table S2. Kinetic fitting parameters of TR-PL data for as-synthesized, PIC- and PyS-passivated samples

Samples	τ_1 (ps)*<br>A_1	τ_2 (ps)<br>A_2
As-syn	0.036 ± 0.001	3.94 ± 0.03
	11.1 ± 0.1	0.65 ± 0.01
PIC	0.033 ± 0.002	4.84 ± 0.04
	7.6 ± 0.1	0.859 ± 0.002
PyS	0.036 ± 0.001	7.08 ± 0.03
	7.0 ± 0.1	0.878 ± 0.002

*The component has a time constant that is significantly faster than the IRF (0.57 ns) of the TR-PL setup, and hence has no physical meaning, but is needed to compensate for the early time kinetics during the fitting.

Table S3. DFT-calculated ligand binding energies for $CsPbX_3$ (X = Cl, Br, I) and a qualitative assessment of defect passivation.

**Ligands**	**$CsPbCl_3$** Binding energy (eV)	**Passivate defects?**	**$CsPbBr_3$** Binding energy (eV)	**Passivate defects?**	**$CsPbI_3$** Binding energy (eV)	**Passivate defects?**
**X = Cl, Br, I**	-2.34	-	-2.01	-	-1.55	-
**OP**	-2.62	Yes	-2.65	Yes	-2.82	No
**PhP**	-2.62	Yes	-2.67	No	-2.75	No
**PyS**	-2.78	Yes	-3.02	No	-2.94	No
**DS**	-2.55	Yes	-2.60	No	-3.03	No
**DBS**	-2.40	Yes	-2.63	Yes	-3.02	No
**Pyr-2-COO**	-1.84	Yes	-1.86	Yes	-2.01	No
**2-Fur**	-2.02	Yes	-1.92	Yes	-2.09	No
**TPC**	-2.04	Yes	-1.92	Yes	-2.06	No
**4-Br-2-TPC**	-2.09	Yes	-2.01	Yes	-2.14	No
**BzO**	-2.31	Yes	-2.20	Yes	-2.31	No
**INC**	-2.15	Yes	-2.16	Yes	-2.24	No
**ANT**	-1.69	Yes	-1.81	Yes	-1.91	No
**PIC**	-2.17	Yes	-2.37	Yes	-2.49	No
**5-Cl-PIC**	-2.38	Yes	-2.29	Yes	-2.42	No
**FUS**	-2.31	Yes	-2.42	No	-2.51	No
**2-ABS**	-2.07	Yes	-1.84	Yes	-1.53	No
**o-ASA**	-1.59	Yes	-1.67	Yes	-1.29	No
**Py**	-0.77	No	-0.79	No	-0.72	Yes
**2-MPy**	-1.38	No	-1.51	No	-1.45	Yes
**PhS**	-0.78	No	-0.87	No	-0.82	Yes
**Th**	-0.43	No	-0.47	No	-0.51	Yes
**3-ABS**	-0.86	No	-1.10	No	-1.40	Yes
**4-ABS**	-0.79	No	-0.95	No	-1.18	Yes

**2-ABA**	-1.15	No	-1.08	No	-0.97	Yes
**PDC**	-1.38	No	-0.86	No	-0.70	Yes
**Cys**	-1.12	No	-1.03	No	-0.97	Yes
**GSH**	-1.06	No	-1.09	No	-1.06	Yes

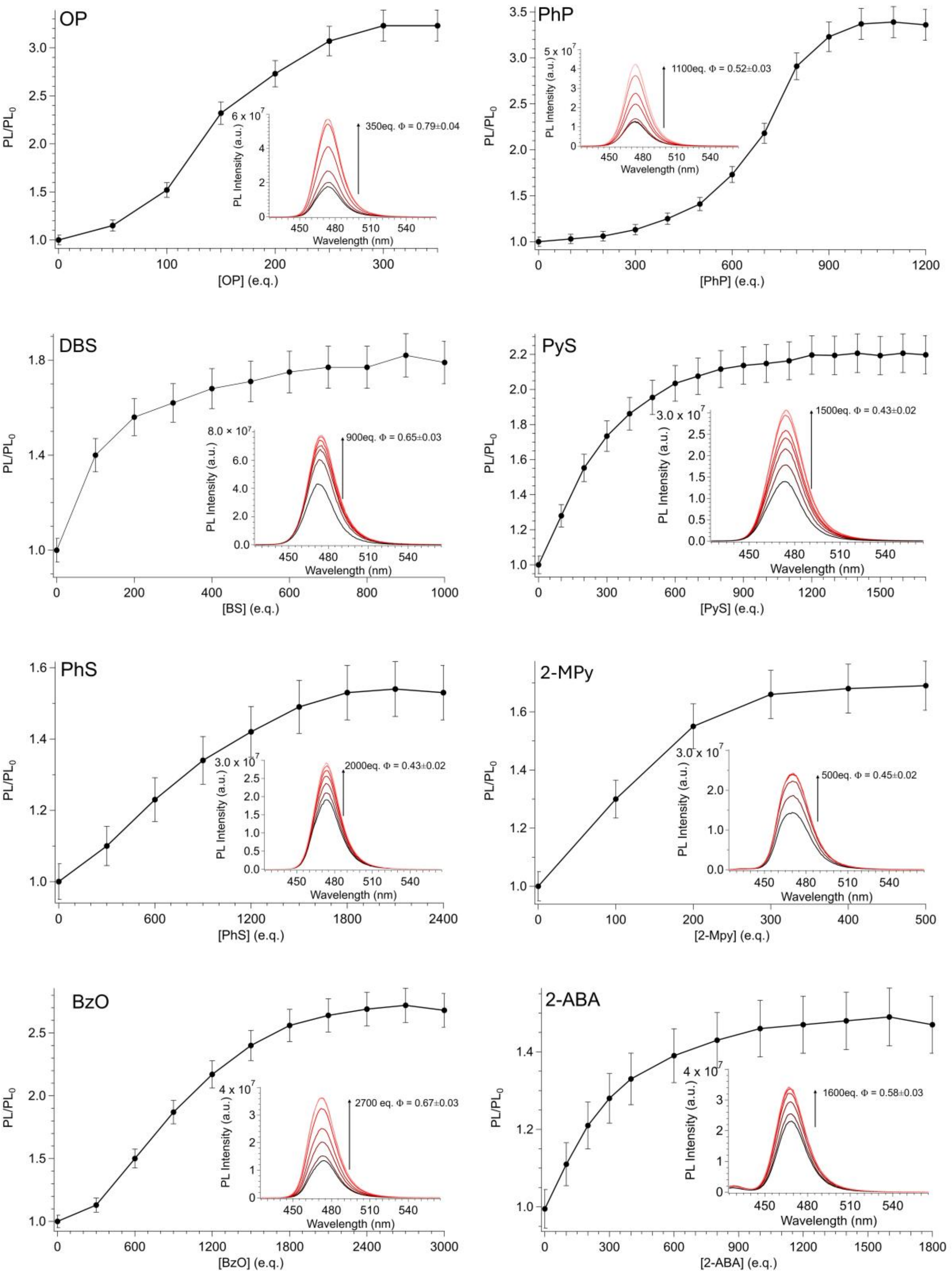

OP
PL/PL0
[OP] (e.q.)
PL Intensity (a.u.)
Wavelength (nm)
350eq. Φ = 0.79±0.04
PhP
[PhP] (e.q.)
1100eq. Φ = 0.52±0.03
DBS
[BS] (e.q.)
900eq. Φ = 0.65±0.03
PyS
[PyS] (e.q.)
1500eq. Φ = 0.43±0.02
PhS
[PhS] (e.q.)
2000eq. Φ = 0.43±0.02
2-MPy
[2-Mpy] (e.q.)
500eq. Φ = 0.45±0.02
BzO
[BzO] (e.q.)
2700 eq. Φ = 0.67±0.03
2-ABA
[2-ABA] (e.q.)
1600eq. Φ = 0.58±0.03

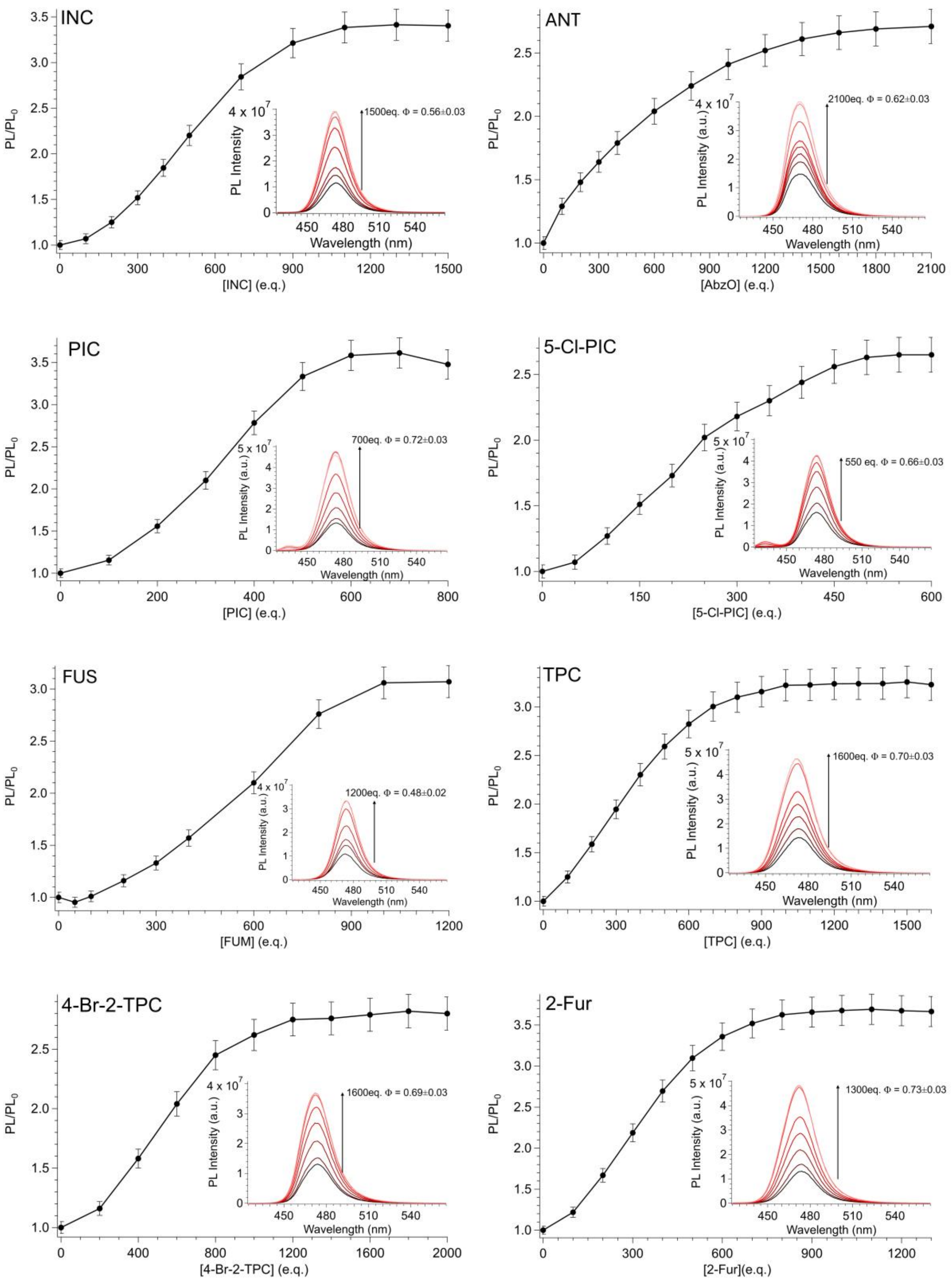
INC
PL/PL$_0$
[INC] (e.q.)
1500eq. Φ = 0.56±0.03
PL Intensity
Wavelength (nm)
ANT
[AbzO] (e.q.)
2100eq. Φ = 0.62±0.03
PL Intensity (a.u.)
PIC
[PIC] (e.q.)
700eq. Φ = 0.72±0.03
5-Cl-PIC
[5-Cl-PIC] (e.q.)
550 eq. Φ = 0.66±0.03
FUS
[FUM] (e.q.)
1200eq. Φ = 0.48±0.02
TPC
[TPC] (e.q.)
1600eq. Φ = 0.70±0.03
4-Br-2-TPC
[4-Br-2-TPC] (e.q.)
1600eq. Φ = 0.69±0.03
2-Fur
[2-Fur](e.q.)
1300eq. Φ = 0.73±0.03

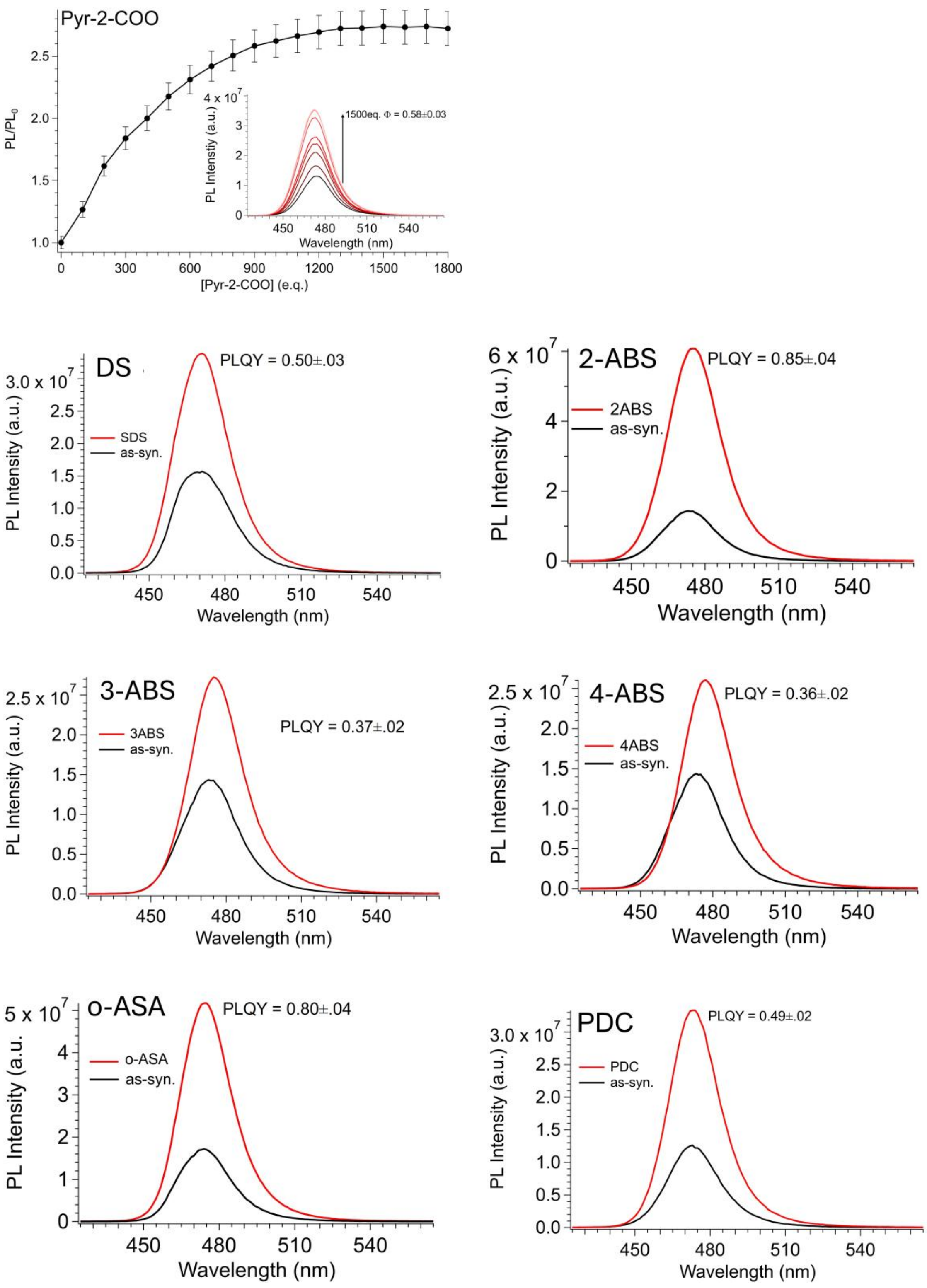

Pyr-2-COO
PL/PL$_0$
[Pyr-2-COO] (e.q.)
1500eq. Φ = 0.58±0.03
PL Intensity (a.u.)
Wavelength (nm)
DS
PLQY = 0.50±.03
SDS
as-syn.
2-ABS
PLQY = 0.85±.04
2ABS
as-syn.
3-ABS
PLQY = 0.37±.02
3ABS
as-syn.
4-ABS
PLQY = 0.36±.02
4ABS
as-syn.
o-ASA
PLQY = 0.80±.04
o-ASA
as-syn.
PDC
PLQY = 0.49±.02
PDC
as-syn.

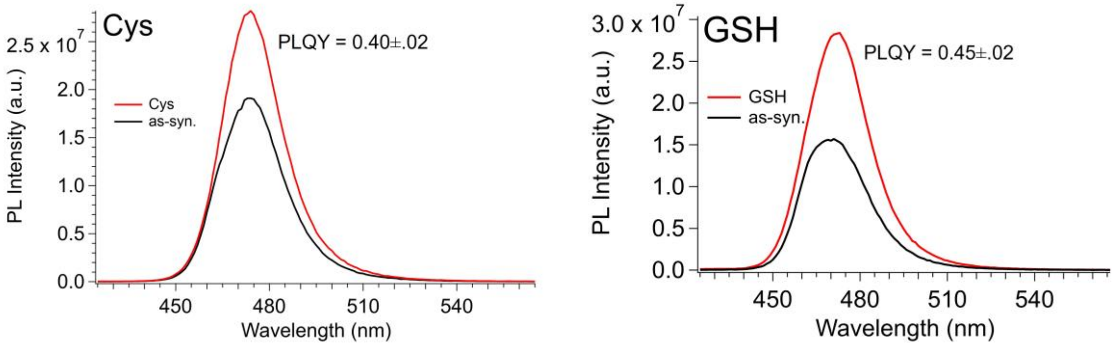

Figure S1. PL spectra and the courses of passivation tests for all ligands. The PLQY of the as-synthesized perovskite nanocrystal varies for different batches of synthesis, and the average value is 0.23 ± 0.06. Ligands using the solid-state exchange procedure were marked as "solid-state", since we did not determine the amounts of ligands introduced to the solution. Other ligands were titrated to the solution as their oleylammonium salts. For ligands applied via the titration method, we provided the increase in PL as a function of the added ligands. For ligands using the solid-state exchange passivation method, we monitored the PLQY change over time, as shown for 2-ABS, and determined that a 16-hour ligand exchange process is sufficient for reaching the equilibrium state. We provided PL spectra for the as-synthesized and the passivated perovskite nanocrystals. Pyridine and thiophene have almost no passivation effect even at high concentration (20,000 e.q.) and were not listed here.

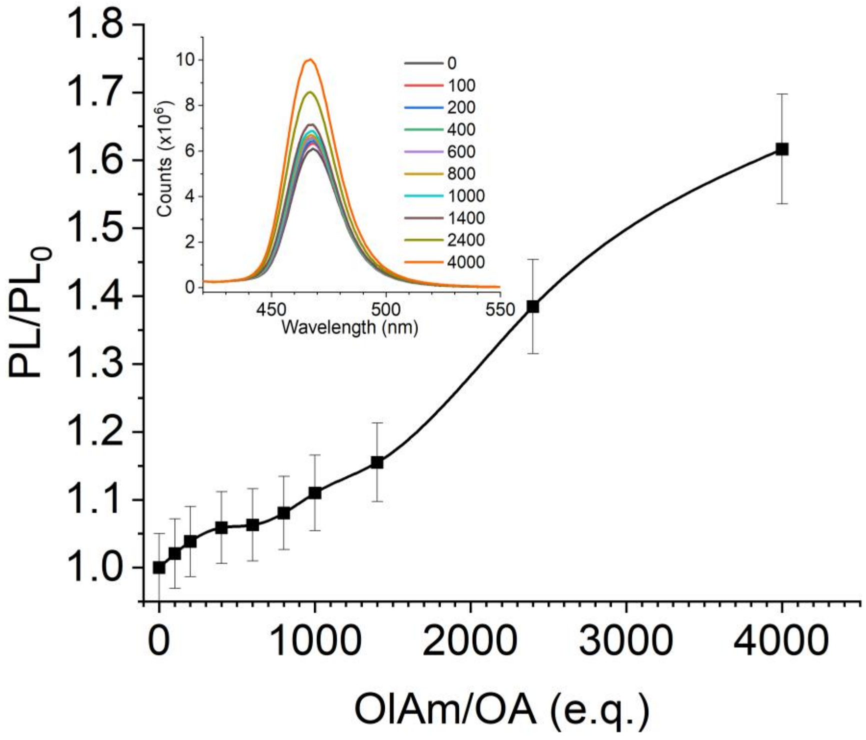

Figure S2. Passivation of the perovskite nanocrystal sample with the OlAm/OA ligand pair. It is known that ammonium can assist in binding weakly bound carboxylate to surface defect sites and improve passivation.[4,7] To assess the effect of extra ammonium on the PLQY during the passivation, we titrated the perovskite nanocrystal sample with the intrinsic synthetic ligand pair, $OlAm^+/OA^-$. With 1500 e.q. OlAm/OA addition, there was only a ~15% increase in PLQY. Even with 4000 e.q. OlAm/OA addition, the PLQY is 0.37±0.03. It must be pointed out that this enhancement was achieved by adding additional OA^-, and therefore, the synergic passivation effect of added $OlAm^+$ should be a minor factor compared to most L-type ligands used in the present study. For instance, 2700 e.q. benzoate/$OlAm^+$ can increase the PLQY to 0.67±0.03. We, therefore, considered that the observed PLQY increases during the passivation test were primarily due to the passivation effect of the chosen ligands.

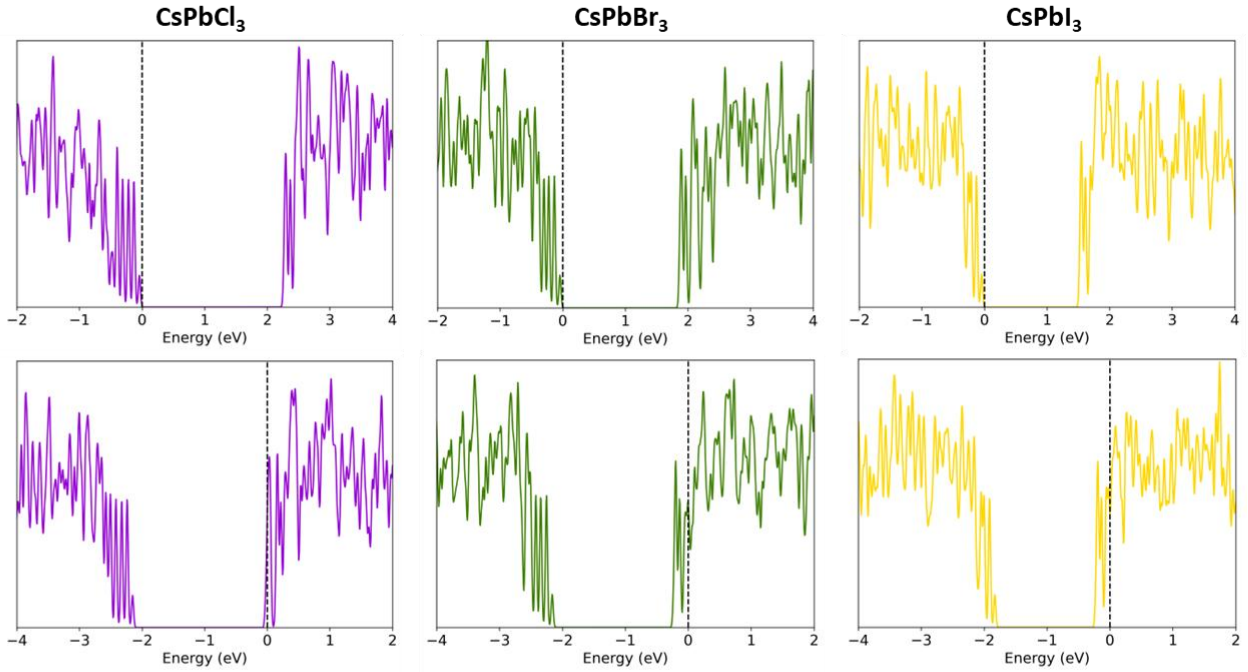

Figure S3. Total density of states (DOS) for $CsPbX_3$ perovskite nanocrystals (X = Cl, Br, I). Top row: defect-free perovskite nanocrystals and bottom row: nanocrystals containing only a surface halide vacancy. The vertical dashed line denotes the Fermi level, set to 0 eV. The defect-free systems exhibit a clean bandgap with the Fermi level located at the valence band maximum (VBM). Upon introduction of a halide vacancy, the Fermi level shifts toward (or into) the conduction band minimum (CBM), consistent with the donor-like character of the vacancy.

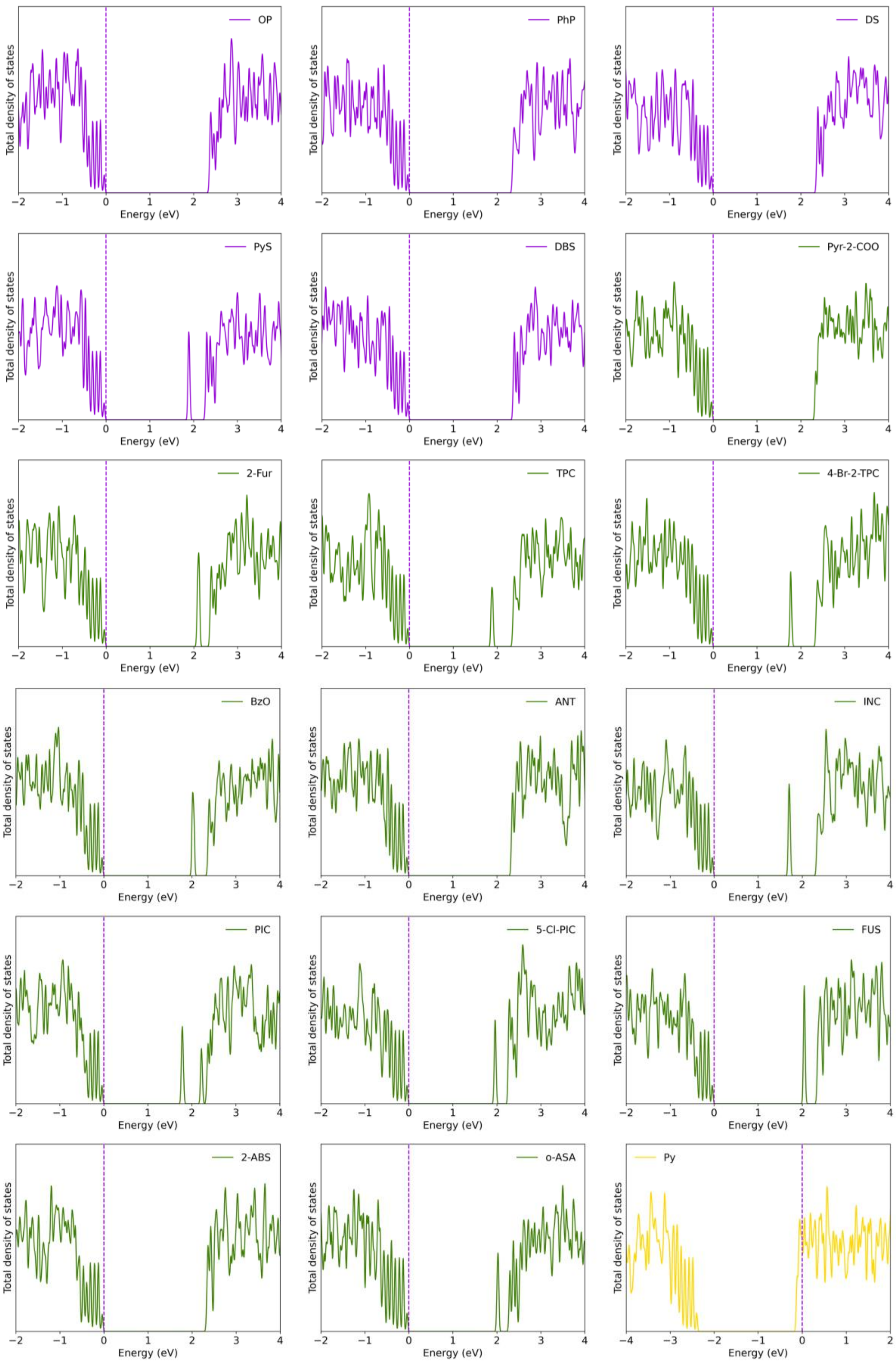
OP
PhP
DS
PyS
DBS
Pyr-2-COO
2-Fur
TPC
4-Br-2-TPC
BzO
ANT
INC
PIC
5-Cl-PIC
FUS
2-ABS
o-ASA
Py
Total density of states
Energy (eV)

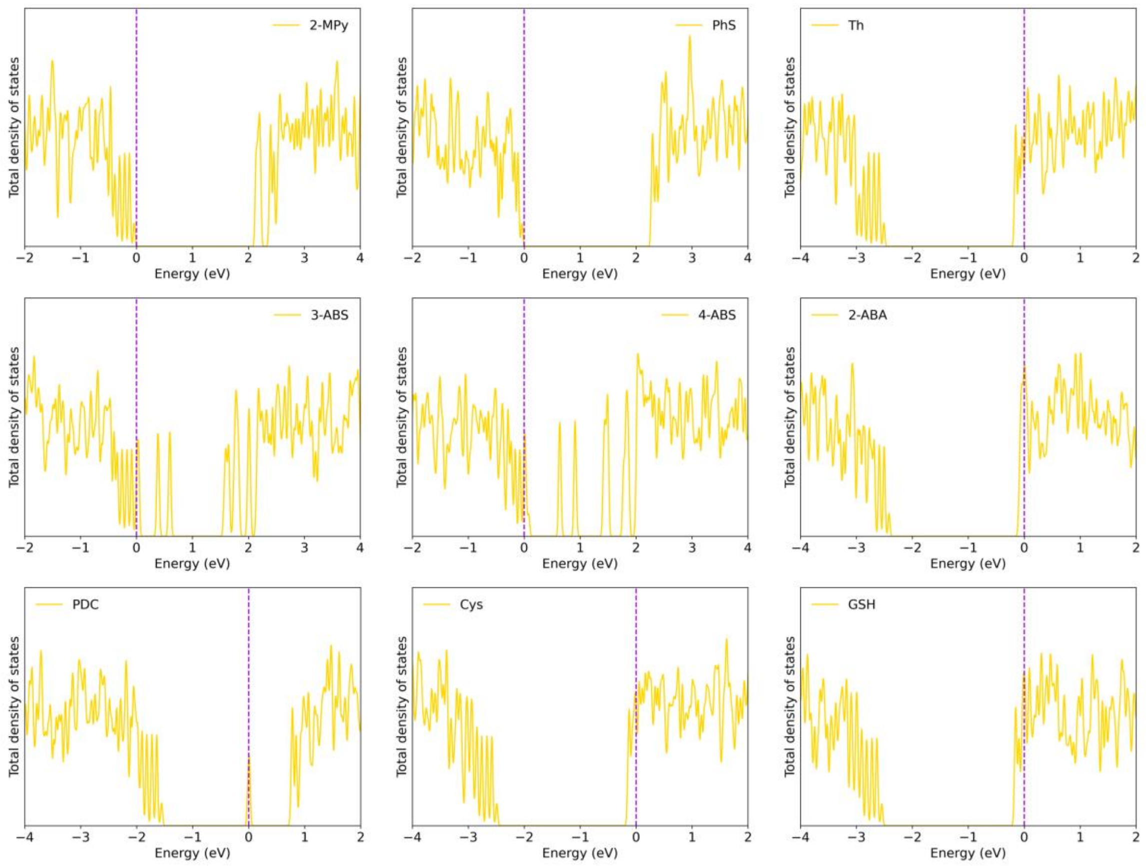

Figure S4. The total density of states (DOS) for ligand-passivated $CsPbCl_3$. The defected state includes a pair of Cs and Cl vacancy (with an additional Cs vacancy for zwitterionic ligands), which are then passivated by MA and the ligand shown in the legend, respectively. The vertical dashed line represents the Fermi level, which has been set to 0 eV.

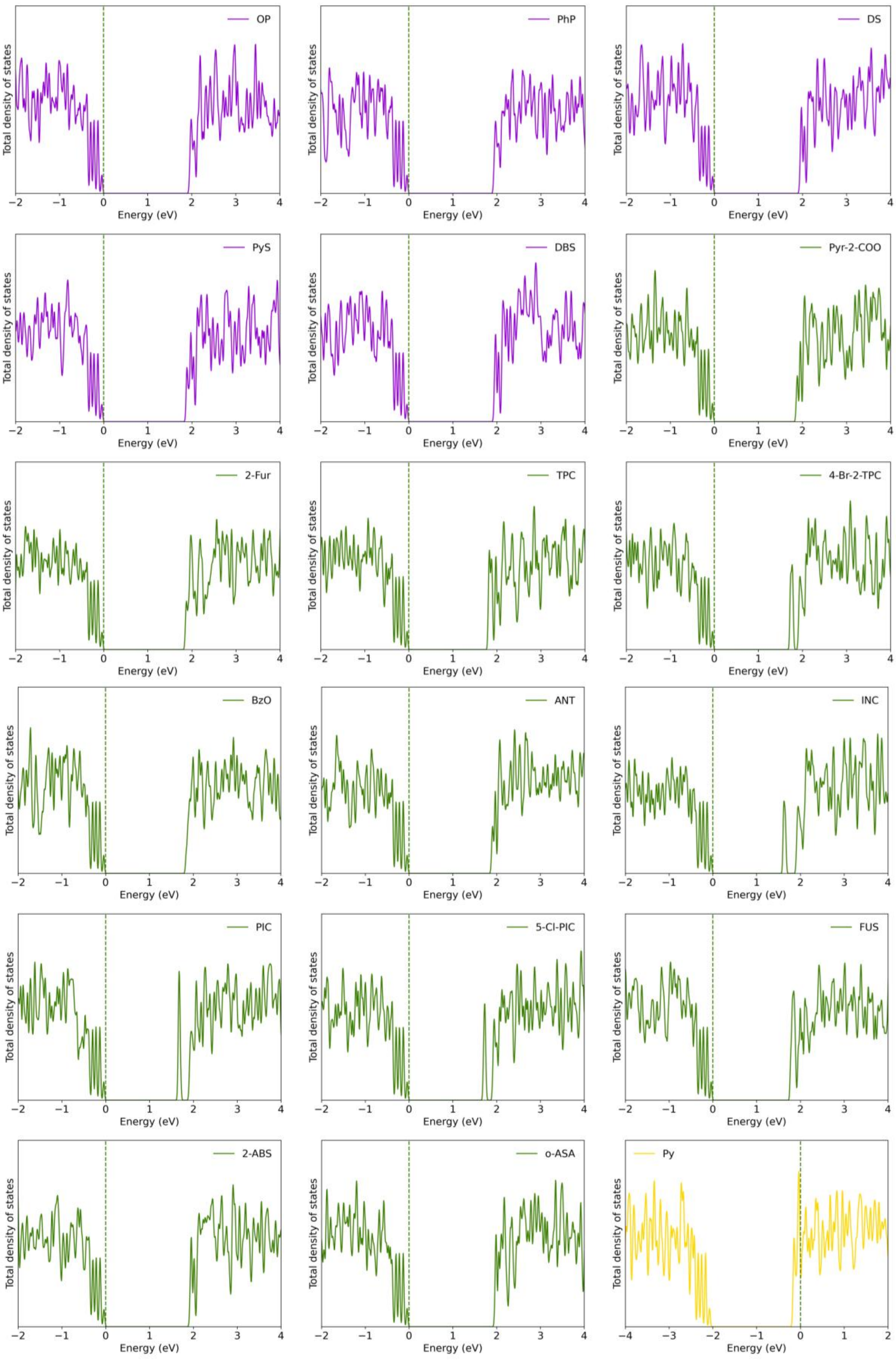

OP
PhP
DS
PyS
DBS
Pyr-2-COO
2-Fur
TPC
4-Br-2-TPC
BzO
ANT
INC
PIC
5-Cl-PIC
FUS
2-ABS
o-ASA
Py
Total density of states
Energy (eV)

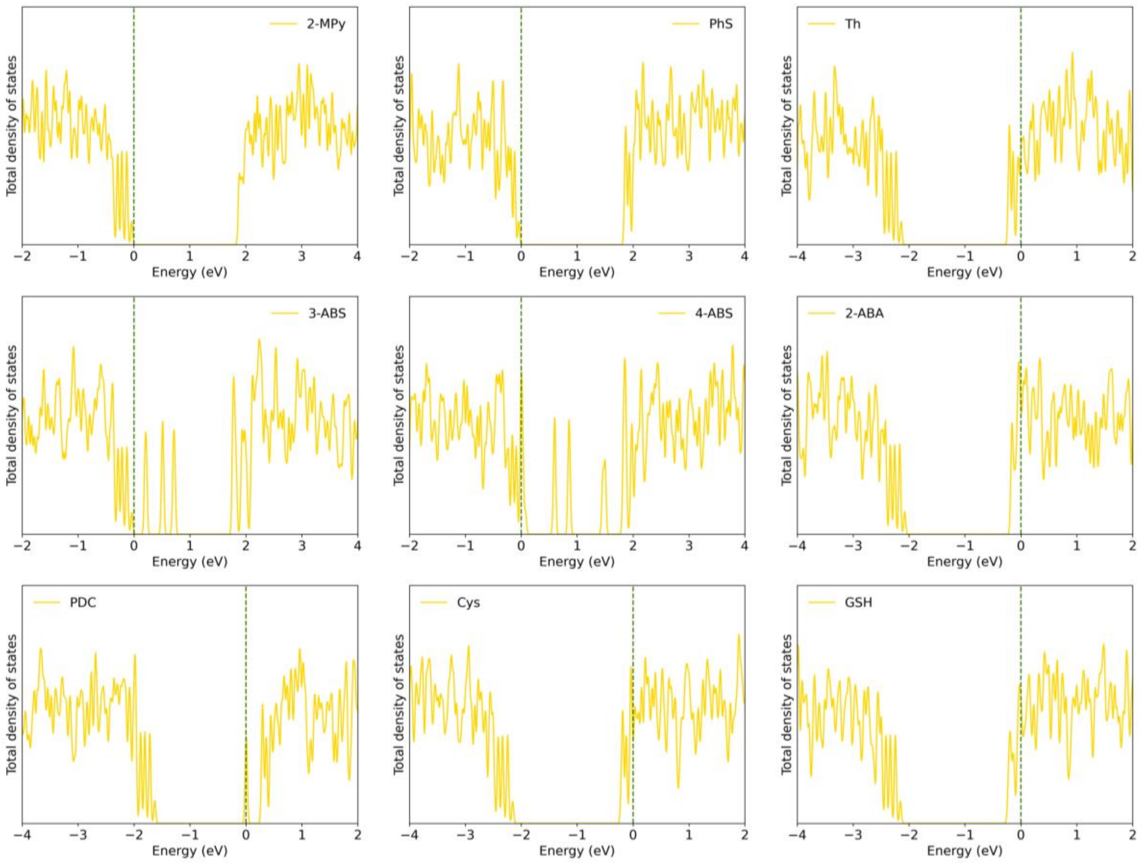

Figure S5. The total density of states (DOS) for ligand-passivated $CsPbBr_3$. The defected state includes a pair of Cs and Br vacancy (with an additional Cs vacancy for zwitterionic ligands), which are then passivated by MA and the ligand shown in the legend, respectively. The vertical dashed line represents the Fermi level, which has been set to 0 eV.

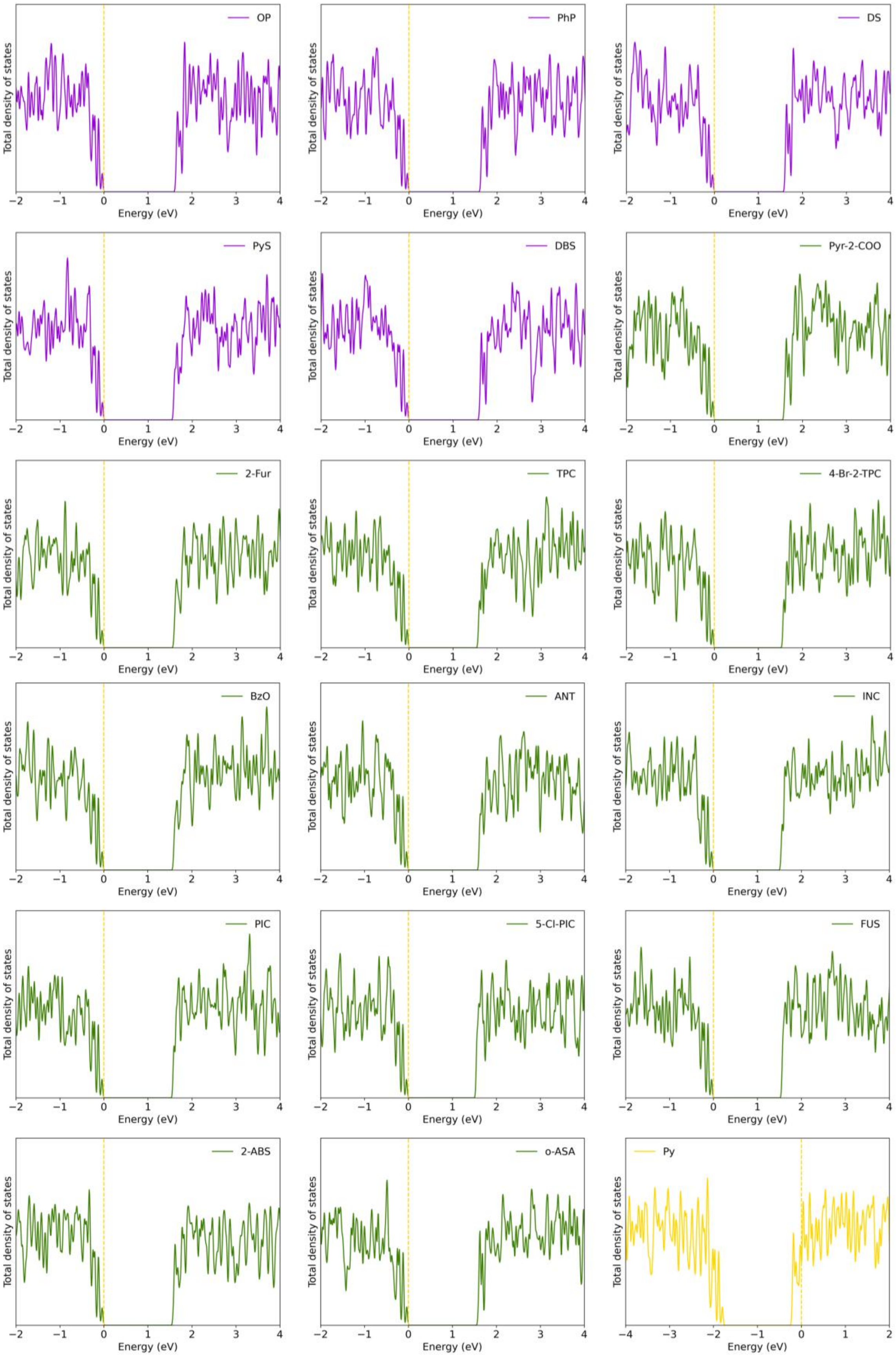

OP
PhP
DS
PyS
DBS
Pyr-2-COO
2-Fur
TPC
4-Br-2-TPC
BzO
ANT
INC
PIC
5-Cl-PIC
FUS
2-ABS
o-ASA
Py
Total density of states
Energy (eV)

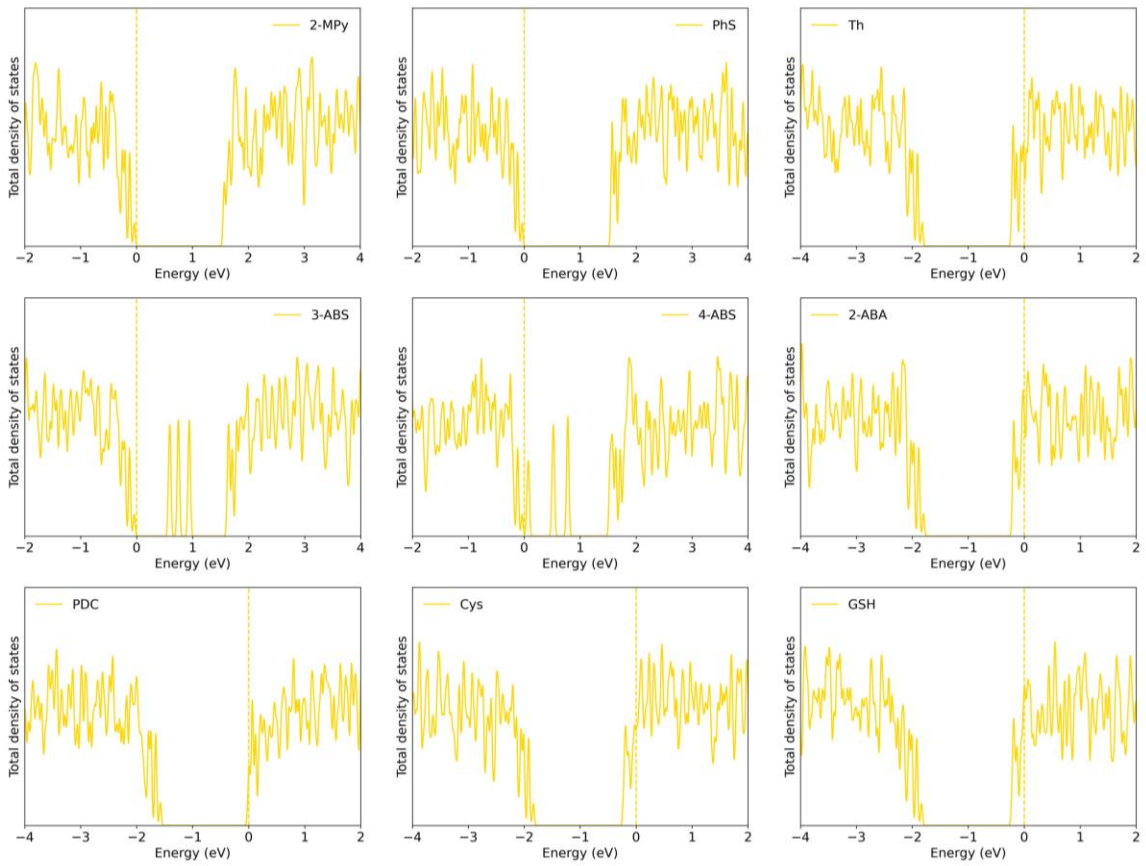

Figure S6. The total density of states (DOS) for ligand-passivated $CsPbI_3$. The defected state includes a pair of Cs and I vacancy (with an additional Cs vacancy for zwitterionic ligands), which are then passivated by MA and the ligand shown in the legend, respectively. The vertical dashed line represents the Fermi level, which has been set to 0 eV.

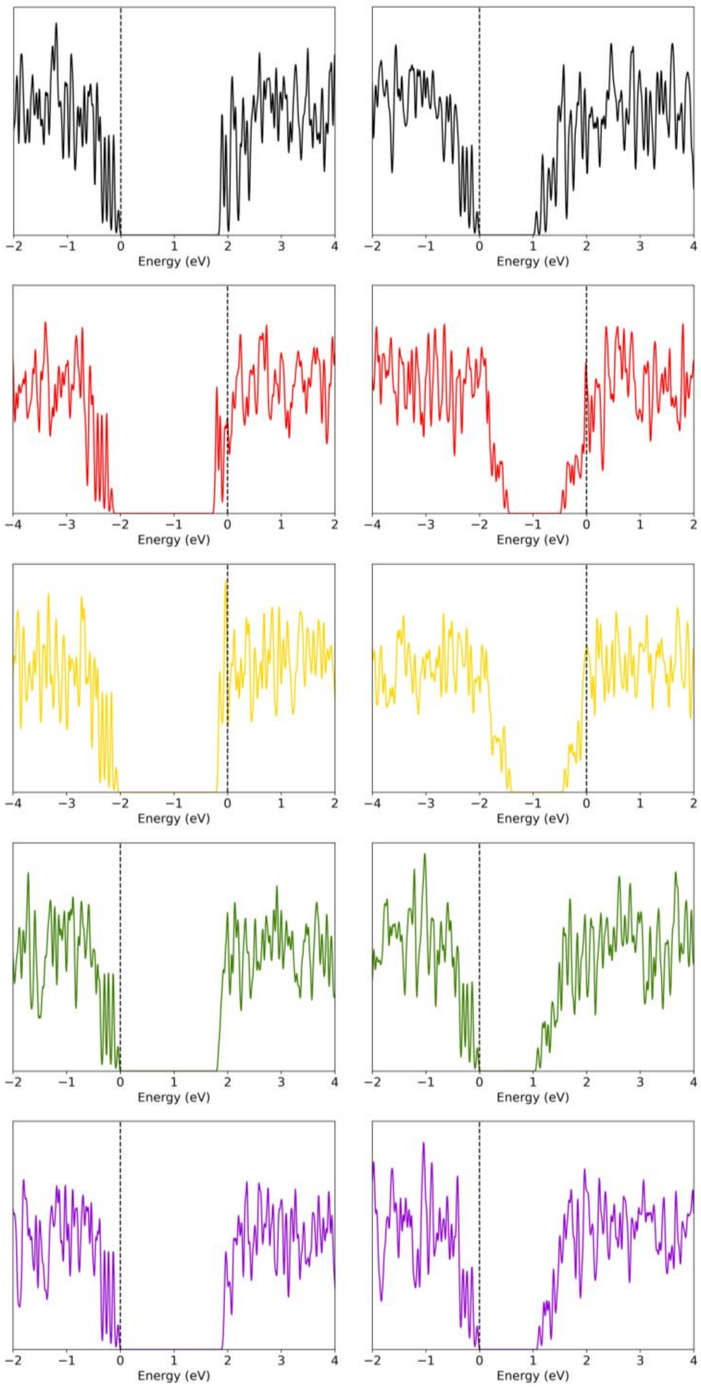

Figure S7. Total density of states (DOS) for $CsPbBr_3$: (a) defect-free, (b) a single halide vacancy, (c) weak-binding ligand (Py)-passivated, (d) medium-binding ligand (BzO)-passivated, and (e) strong-binding ligand (PhP)-passivated systems. The left column shows DOS calculated without spin-orbit coupling (SOC), and the right column shows DOS including SOC. The vertical dashed line denotes the Fermi level, set to 0 eV. Inclusion of SOC reduces the magnitude of the bandgap, but the relative Fermi level positions and the overall trends in ligand passivation remain unchanged.

References

(1) ten Brinck, S.; Zaccaria, F.; Infante, I. Defects in Lead Halide Perovskite Nanocrystals: Analogies and (Many) Differences with the Bulk. *ACS Energy Lett.* **2019**, *4* (11), 2739–2747. https://doi.org/10.1021/acsenergylett.9b01945.

(2) Chen, Y.; Smock, S. R.; Flintgruber, A. H.; Perras, F. A.; Brutchey, R. L.; Rossini, A. J. Surface Termination of CsPbBr3 Perovskite Quantum Dots Determined by Solid-State NMR Spectroscopy. *J. Am. Chem. Soc.* **2020**, *142* (13), 6117–6127. https://doi.org/10.1021/jacs.9b13396.

(3) Kang, J.; Wang, L.-W. High Defect Tolerance in Lead Halide Perovskite CsPbBr3. *J. Phys. Chem. Lett.* **2017**, *8* (2), 489–493. https://doi.org/10.1021/acs.jpclett.6b02800.

(4) Nenon, D. P.; Pressler, K.; Kang, J.; Koscher, B. A.; Olshansky, J. H.; Osowiecki, W. T.; Koc, M. A.; Wang, L.-W.; Alivisatos, A. P. Design Principles for Trap-Free CsPbX3 Nanocrystals: Enumerating and Eliminating Surface Halide Vacancies with Softer Lewis Bases. *J. Am. Chem. Soc.* **2018**, *140* (50), 17760–17772. https://doi.org/10.1021/jacs.8b11035.

(5) Malinoski, A.; Hu, G.; Wang, C. Strong Bidentate Coordination for Surface Passivation and Ligand-Shell Engineering of Lead Halide Perovskite Nanocrystals in the Strongly Quantum-Confined Regime. *J. Phys. Chem. C* **2021**, *125* (44), 24521–24530. https://doi.org/10.1021/acs.jpcc.1c07952.

(6) ten Brinck, S.; Infante, I. Surface Termination, Morphology, and Bright Photoluminescence of Cesium Lead Halide Perovskite Nanocrystals. *ACS Energy Lett.* **2016**, *1* (6), 1266–1272. https://doi.org/10.1021/acsenergylett.6b00595.

(7) De Roo, J.; Ibáñez, M.; Geiregat, P.; Nedelcu, G.; Walravens, W.; Maes, J.; Martins, J. C.; Van Driessche, I.; Kovalenko, M. V.; Hens, Z. Highly Dynamic Ligand Binding and Light Absorption Coefficient of Cesium Lead Bromide Perovskite Nanocrystals. *ACS Nano* **2016**, *10* (2), 2071–2081. https://doi.org/10.1021/acsnano.5b06295.

(8) Ravi, V. K.; Santra, P. K.; Joshi, N.; Chugh, J.; Singh, S. K.; Rensmo, H.; Ghosh, P.; Nag, A. Origin of the Substitution Mechanism for the Binding of Organic Ligands on the Surface of CsPbBr3 Perovskite Nanocubes. *J. Phys. Chem. Lett.* **2017**, *8* (20), 4988–4994. https://doi.org/10.1021/acs.jpclett.7b02192.

(9) Krieg, F.; Ochsenbein, S. T.; Yakunin, S.; ten Brinck, S.; Aellen, P.; Süess, A.; Clerc, B.; Guggisberg, D.; Nazarenko, O.; Shynkarenko, Y.; Kumar, S.; Shih, C.-J.; Infante, I.; Kovalenko, M. V. Colloidal CsPbX3 (X = Cl, Br, I) Nanocrystals 2.0: Zwitterionic Capping Ligands for Improved Durability and Stability. *ACS Energy Lett.* **2018**, *3* (3), 641–646. https://doi.org/10.1021/acsenergylett.8b00035.

(10) Wang, C.; Malinoski, A.; Yuan, J.; Brea, C.; Hu, G. A Surface Engineering Approach for Promoting Dexter Energy Transfer from Lead Halide Perovskite Nanocrystals. *J. Phys. Chem. C* **2023**, *127* (2), 1135–1144. https://doi.org/10.1021/acs.jpcc.2c07664.
